# Sensory cells in tunicates: insights into mechanoreceptor evolution

**DOI:** 10.3389/fcell.2024.1359207

**Published:** 2024-03-14

**Authors:** Chiara Anselmi, Gwynna K. Fuller, Alberto Stolfi, Andrew K. Groves, Lucia Manni

**Affiliations:** ^1^ Hopkins Marine Station, Institute for Stem Cell Biology and Regenerative Medicine, Stanford University, Pacific Grove, CA, United States; ^2^ Wu Tsai Neurosciences Institute, Stanford University, Stanford, CA, United States; ^3^ Department of Molecular and Human Genetics, Baylor College of Medicine, Houston, TX, United States; ^4^ School of Biological Sciences, Georgia Institute of Technology, Atlanta, GA, United States; ^5^ Department of Neuroscience, Baylor College of Medicine, Houston, TX, United States; ^6^ Dipartimento di Biologia, Università degli Studi di Padova, Padova, Italy

**Keywords:** mechanoreceptor, evolution, placode, chordates, hair cells, primary sensory cells, secondary sensory cells

## Abstract

Tunicates, the sister group of vertebrates, offer a unique perspective for evolutionary developmental studies (Evo-Devo) due to their simple anatomical organization. Moreover, the separation of tunicates from vertebrates predated the vertebrate-specific genome duplications. As adults, they include both sessile and pelagic species, with very limited mobility requirements related mainly to water filtration. In sessile species, larvae exhibit simple swimming behaviors that are required for the selection of a suitable substrate on which to metamorphose. Despite their apparent simplicity, tunicates display a variety of mechanoreceptor structures involving both primary and secondary sensory cells (*i.e*., coronal sensory cells). This review encapsulates two decades of research on tunicate mechanoreception focusing on the coronal organ’s sensory cells as prime candidates for understanding the evolution of vertebrate hair cells of the inner ear and the lateral line organ. The review spans anatomical, cellular and molecular levels emphasizing both similarity and differences between tunicate and vertebrate mechanoreception strategies. The evolutionary significance of mechanoreception is discussed within the broader context of Evo-Devo studies, shedding light on the intricate pathways that have shaped the sensory system in chordates.

## 1 Introduction

**FIGURE 1:**
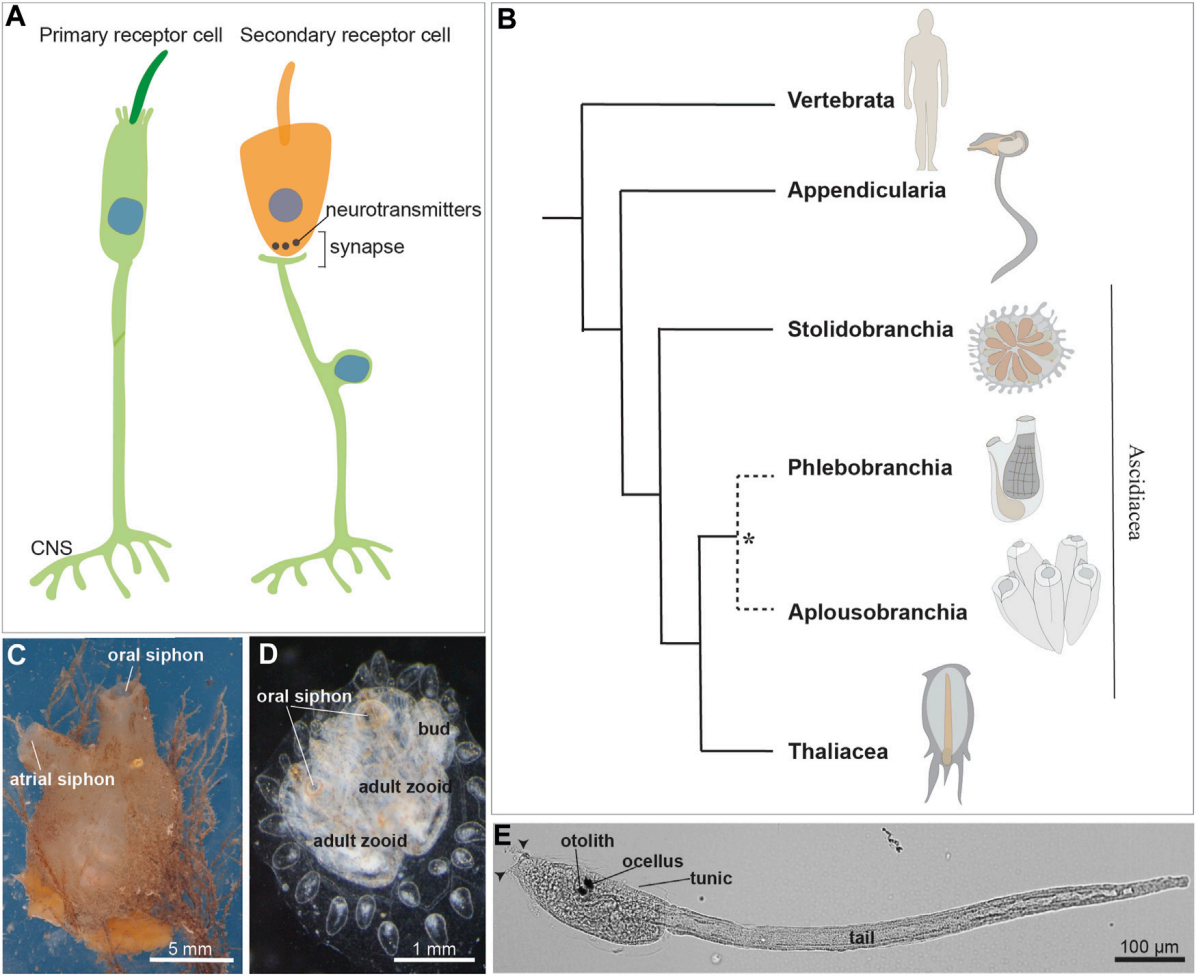
**(A)** Illustration of a primary and a secondary receptor cell in tunicates. The primary receptor is a peripheral neuron, whose soma (indicated by the blue nucleus) is in the epidermis. The secondary receptor (orange) is, *vice versa*, a dedicated receptor that transmits its input to a brain sensory neuron. **(B)** Chordate evolutionary tree. * The monophyly of Phlebobranchia is disputed [see (DeBiasse et al., 2020)]. Stolidobranchia species are defined as Pleurogona (with gonads in the lateral body wall), whereas Phlebobranchia and Aplousobranchia are defined as Enterogona (with gonads close to the gut). **(C)** Adult individual of the ascidian *Molgula socialis* (right view). **(D)** Young colony of *Botryllus schlosseri* composed by two adult zooids and their buds. Dorsal view. **(E)** *Ciona robusta* larva at stage 28, 18 h post fertilization at 20°. Arrowheads: two anterior papillae. Ascidian larvae are composed of an anterior cephalenteron, *i.e.*, a body part including both head structures (such as the brain) and trunk structures (such as the gut), and a posterior tail. The cephalenteron is usually called a “trunk”.

Twenty years ago, a paper provocatively titled “Novel, secondary sensory cell organ in ascidians: in search of the ancestor of the vertebrate lateral line” by Burighel and others (Burighel et al., 2003), provided evidence that the tunicate ascidian *Botryllus schlosseri* possessed a complex mechanosensory organ, the coronal organ. Unlike the previously characterized multicellular mechanoreceptor organs of adult tunicates (Manni and Pennati, 2015), this novel organ was not composed of peripheral neurons (*i.e.*, primary sensory cells) but showed dedicated axonless secondary receptor cells. These secondary receptor cells were contacted at their base by neurites coming from brain neurons, forming both afferent and efferent synapses with the sensory cells (Figure 1A). This discovery also revealed that the adult tunicate brain possessed sensory neurons, since then not considered, for the elaboration of afferent information from the coronal sensory cells and their control by means of efferent inputs. Moreover, in *B. schlosseri*, coronal sensory cells showed an apical bundle with a cilium accompanied by microvilli and/or stereovilli. They were aligned on the oral siphon tentacles and exposed to the incoming seawater. In many aspects, these cells resembled vertebrate hair cells of lateral line organs (Manni et al., 2004). These features, combined with the evolutionary proximity between tunicates and vertebrates, considered sister groups (Delsuc et al., 2018), initiated a controversial yet exciting debate on the homology of coronal sensory cells and hair cells. This discussion extended to the homology of the embryonic territories from which they originate. Vertebrate hair cells derive from neurogenic placodes (namely, from the otic and the lateral line placodes) that, together with the neural crest, were at that time considered exclusive to vertebrates (Manni et al., 2001; 2004). Therefore, the discovery of the coronal organ (with sensory cells hypothesized homologous to vertebrate hair cells), together with the publication of the first data on the presence of placodal area and neural crest-like cells in tunicates (Manni et al., 2001; Jeffery et al., 2004; Mazet and Shimeld, 2005), challenged the foundation of the main theory of vertebrate evolution, the so-called “New head hypothesis” (Gans and Northcutt, 1983). This theory proposed that neurogenic placodes and neural crest cells were, with respect to non-vertebrate chordates, novel cell populations that contributed to the success of vertebrates and their development of complex nervous systems.

In the past 20 years since the discovery of the coronal organ, numerous aspects of its morphology, physiology, and development have been elucidated (Burighel et al., 2011; Manni and Pennati, 2015). The organ has been found in all the tunicate taxa (except for Salps, see below) so far examined. Its mechanoreceptive function and synaptic connectivity has been established and some key developmental genes studied. Nevertheless, many questions remain unanswered, making the investigation of tunicate mechanoreception an intriguing question in evolutionary developmental biology research.

Tunicates constitute a diverse group of marine invertebrates, including both pelagic and sessile animals with different behavior and motility, thus having varied sensory requirements. Traditionally, tunicates were classified into three classes: the sessile Ascidiacea and the pelagic Thaliacea and Larvacea. However, molecular phylogenies suggest that ascidians are a paraphyletic group and support the monophyly of thaliaceans (Delsuc et al., 2018; Kocot et al., 2018; DeBiasse et al., 2020) (Figures 1B–D). The tunicate tadpole swimming larva exhibits a typical chordate body plan which is lost during metamorphosis in ascidians and thaliaceans. At this stage the latter adopts a sac-like body with two apertures, the oral and the atrial siphons, for seawater circulation and filtration. The sessile ascidians, the most extensively studied group, exhibit a larva with a tripartite brain derived from the dorsal nerve tube. The larva also possesses numerous primary mechanosensory cells scattered in the monolayered epidermis, allowing the detection of a suitable substrate for metamorphosis (Wakai et al., 2021; Sakamoto et al., 2022). The sessile adult has a ganglionic brain, and its mobility is limited to the siphon and body wall contraction as defensive responses (Mackie and Burighel, 2005).

Here we review research on mechanoreception in tunicates. We begin by examining mechanoreceptor cells and organs based on primary receptors in the ascidian larva (Section 2). Then, we describe the diversity of mechanoreception structures (including both single or clustered cells and multicellular organs) based on primary receptors exhibited by adult tunicates (Section 3). Lastly, we consider 20 years of research on the coronal organ from a morphological, physiological and developmental point of view, describing similarities and differences between coronal sensory cells and vertebrate hair cells (Section 4).

## 2 Putative mechanosensory cells of the ascidian larva

**FIGURE 2:**
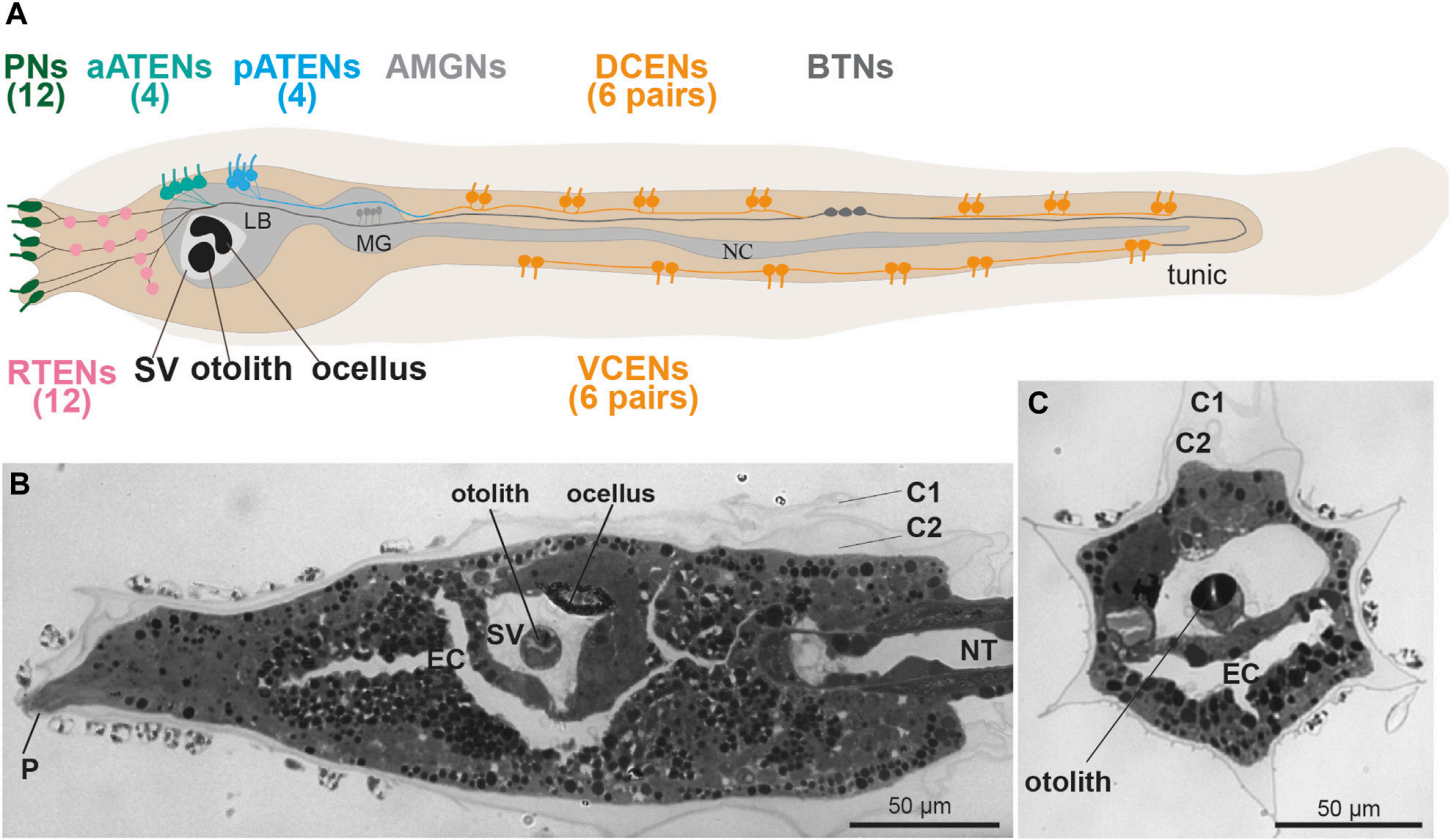
**(A)** Larval sensory neurons in *Ciona robusta* (see Ryan et al., 2017). Left lateral view. In the tail there are two groups of caudal epidermal neurons, 12 dorsal (DCENs) and 12 ventral (VCENs). There are three groups of Trunk Epidermal Neurons, 12 rostral (RTEN), four anterior (aATENs) and four posterior (pATENs). There are three rostral papillae with two pairs of sensory neurons (Pns) each. **(B,C)** frontal and cross sections of *C. robusta*, showing some sensory structures: the anterior dorsal papilla (P), and the sensory vesicle (SV) with the otolith and the ocellus, Toluidine blue. C1-C2: tunic cuticular layer; EC: endodermal cavity; LB: larval brain; MG: motor ganglion; NC: nerve cord; NT: notochord; SV: sensory vesicle.

As the larvae are primarily responsible for ascidian dispersal, their simple swimming behavior is modified by environmental stimuli. This is likely to increase the odds of escaping predation and settling in a suitable location for metamorphosis. For instance, mechanical stimulation of the adhesive/sensory papillae, the three sensory organs (two dorsal and one ventral) located in the anterior larval region (Figures 1E, 2A–B) is sufficient and necessary to trigger metamorphosis in *Ciona* (Wakai et al., 2021; Sakamoto et al., 2022). While mechanosensitive modulation of swimming has yet to be definitively shown in ascidian larvae, startle-like behaviors have been described in *Ciona* (Athira et al., 2022). Here we discuss what is known about the development and function of the candidate primary mechanosensory cell types that have been identified in these larvae.

### 2.1 Caudal epidermal neurons

Sometimes referred to as Caudal Epidermal Sensory Neurons, these primary receptors are a subset of a broader class of tail epidermal neurons and were initially described in *Diplosoma listerianum* (previously named *D. macdonaldi*) (Torrence and Cloney, 1982). They have since been reported in numerous other species spread across both enterogonid (e.g., *Ciona, Phallusia*) and stolidobranch (e.g., *Halocynthia, Molgula*) ascidians (Torrence and Cloney, 1982; Takamura, 1998; Imai and Meinertzhagen, 2007; Terakubo et al., 2010; Ohtsuka et al., 2014; Ryan et al., 2018). Although the CENs have yet to be conclusively shown to be mechanosensory, Torrence and Cloney proposed this based on ultrastructural similarities to cupular organ mechanoreceptors of the adult (Torrence and Cloney, 1982). (Figure 1E) CENs are found embedded in the epidermis of the larval tail, along both the dorsal and ventral midlines (Figure 1E). Therefore they are sometimes divided into Dorsal CEN (DCEN) and Ventral CEN (VCEN) subtypes (Ryan et al., 2018) (Figure 2A). CENs occur in regularly interspersed pairs, but their number is variable, with an average of 14 pairs per larva reported in *Ciona* (Pasini et al., 2006). Each neuron bears a single cilium that projects into the overlying extracellular tunic. In the tunic these cilia form a branched network termed the ASNET (Ascidian Dendritic Network In Tunic) (Torrence and Cloney, 1982; Konno et al., 2010; Terakubo et al., 2010; Yokoyama et al., 2014). While the base of the cilia are formed by microtubules and are clearly stained by anti-acetylated tubulin immunofluorescence, their distal portions in the tunic do not contain ordered microtubule arrays (Torrence and Cloney, 1982; Terakubo et al., 2010). CENs possess short axons that form contacts primarily between each other and a few putative relay neurons, such as the Bipolar Tail Neurons (BTNs) (Stolfi et al., 2015; Ryan et al., 2018). The BTNs have been proposed to be homologous to vertebrate cranial sensory neurons (Papadogiannis et al., 2022), though their sensory capabilities are entirely unknown. Like all epidermal neurons, CENs express *Slc17a6/7/8,* encoding Vesicular glutamate transporter (Vglut) and are therefore likely glutamatergic (Horie et al., 2008).

Extensive work in *Ciona* has revealed the embryonic origins of the CENs and the molecular pathways regulating their specification. CENs arise from neurogenic midlines of the tail epidermis, both dorsally and ventrally. Both midlines are derived from b-lineage blastomeres that also give rise to epidermal cells and BTNs (Pasini et al., 2006). While induction of the dorsal and ventral neurogenic midlines occurs independently through different signals, they converge on a shared gene regulatory network for sensory neurogenesis (Pasini et al., 2006; 2012; Tang et al., 2013; Waki et al., 2015). Both midlines express Msx, which in turn activates the expression of the proneural bHLH gene, *Achaete-Scute-like.a* (*Ascl.a,* though sometimes referred to as *Ascl2* or *Ascl.b* previously)*.* However, in the dorsal midline, *Msx* is activated by Otx and Nodal, while in the ventral midline it is activated instead by Tbx2/3 (Pasini et al., 2006; Waki et al., 2015). Otx and Nodal expression in the dorsal midline in turn depends on FGF signaling, while Tbx2/3 expression in the ventral midline is induced by ADMP/BMP signaling instead (Pasini et al., 2006; Waki et al., 2015). Downstream of Ascl.a, both dorsal and ventral networks appear to function through a series of transcription factors, especially conserved neuronal selectors such as Pou4 and Myt1 (Tang et al., 2013). While all the cells in these neurogenic midlines express Ascl.a and thus likely have the potential to give rise to CENs, the final number of neurons is limited by typical lateral inhibition via the Delta/Notch pathway and the microRNA miR-124 (Chen et al., 2011; Tang et al., 2013).

Given that the dorsal and ventral neurogenic midlines are induced by different mechanisms, it has been proposed that one may have evolved as a co-option of the other (Waki et al., 2015). More specifically, it was proposed that the ventral midline is the ancestral one, as induction of ventrolateral sensory neurons by BMP is observed in cephalochordates as well (Lu et al., 2012). The neurogenic dorsal midline of tunicates and vertebrates would therefore represent a co-option of this neurogenic program in the last common olfactorian ancestor (last common ancestor of tunicates and vertebrates). In vertebrates, the dorsal neurogenic domain would have allowed for the emergence of neural crest-derived neurons and other sensory neuron types, like Rohon-Beard cells of anamniote larvae. Alternatively, the ventral midline may have evolved specifically in tunicates as a co-option of an ancestral Msx-dependent neural plate border program for sensory neuron specification. Complementary to these scenarios, it has also been suggested that both dorsal and ventral midlines were neurogenic in the chordate ancestor, and that vertebrates lost the ventral one (Pasini et al., 2006). However, it was reported that another tunicate species, *Halocynthia roretzi,* has only a small number of ventral CENs near the tail tip, and that its dorsal neurogenic midline depends on FGF, Nodal, and BMP combined, along with yet-undiscovered inductive signals (Ohtsuka et al., 2014). Although the midline neurogenic programs are deeply conserved across tunicates (Coulcher et al., 2020), it is clear that additional work on diverse tunicate species will be required to better refine our evolutionary models.

### 2.2 Trunk epidermal neurons (RTENs, aATENs, and pATENs)

In addition to putative mechanosensory neurons of the tail, there are three epidermal neuron subtypes found in the dorsal areas of the epidermis of the larval “trunk”. These primary receptors were defined as Trunk Epidermal Neurons. These are, from anterior to posterior, Rostral Trunk Epidermal Neurons (RTENs), Anterior Apical Trunk Epidermal Neurons (aATENs), and Posterior Trunk Epidermal Neurons (pATENs) (Imai and Meinertzhagen, 2007; Ryan et al., 2018) (Figure 2A). In *Ciona*, there are 7 RTENs on either left/right side (14 total neurons) of the dorsolateral epidermis between the papillae and the sensory vesicle (Ryan et al., 2018). The aATENs occur as two left/right pairs on either side of the dorsal midline (4 total neurons), while the four pATENs appear to lie directly on the midline (Ryan et al., 2018). Although all trunk epidermal neurons contribute to the larval ASNET, like CENs their mechanosensory abilities have never been tested (Abitua et al., 2015; Poncelet et al., 2022; Hoyer et al., 2024). The three subtypes all have well-developed axons but show different connectivity within the PNS network, hinting at distinct functions. RTENs form extensive chemical synapses onto a few different interneurons in the larval brain including the Eminens cells, which are GABAergic (Cao et al., 2019). pATENs on the other hand form extensive inputs onto the Ascending Motor Ganglion (AMG) complex, especially the sole cholinergic (i.e., excitatory) AMG neuron, AMG5 (Ryan et al., 2018; Ryan et al., 2016; Ryan et al., 2017; Kourakis et al., 2019; Popsuj and Stolfi, 2021). Downstream connections even suggest opposite effects on swimming behavior, either arresting swimming (RTENs) or triggering swimming (pATENs). In contrast, the aATENs do not appear to form very many chemical synapses, at least at the relatively early larval stage documented by the connectome studies (Ryan et al., 2018). This may support its proposed role as a neurosecretory cell, and potentially homologous to both olfactory neurons and Gonadotropin-releasing hormone (GnRH) neurons in vertebrates (Abitua et al., 2015; Okawa et al., 2020).

Much less is known about the development of the different Trunk Epidermal Neurons, compared to the CE Ns. In *Halocynthia,* RTENs are specified from anterior neural plate lateral border cells by FGF, Nodal, and BMP signaling (Ohtsuka et al., 2014), while excess BMP signaling appears to suppress the formation of the oral siphon placode, or stomodeum, which gives rise to the aATENs in Ciona (Abitua et al., 2015). A similar FGF/Nodal/BMP combination is required for CEN specification in Halocynthia (see above), though this may be different in *Ciona* and other tunicate families. This suggests that a common gene regulatory network might be shared between Trunk and Caudal subsets of ESNs. The development of the pATENs has not been studied at all, to our knowledge. In sum, much work remains to be done on both the function and development of these different Trunk Epidermal Neurons.

### 2.3 Papilla neurons

Despite our current knowledge of the *Ciona* larval connectome and the regulation of caudal and Trunk Epidermal Neuron development, there is little direct evidence supporting their mechanosensitive nature. There is no evidence directly refuting that CENs and assorted Trunk Epidermal Neurons are mechanosensory cells, either. However, the larval neuron most widely accepted as a mechanosensitive cell type is the Papilla Neuron (PN) (Figure 2A) (Manni et al., 2021). In *Ciona,* PNs (sometimes called Papilla Sensory Neurons or Primary Sensory Neurons of the Papillae) are found surrounding the three adhesive/sensory papillae at the very anterior end of the larva (Zeng et al., 2019). There are two dorsal papillae (one left, one right) and one medial ventral papilla (Figures 2A, B). Each papilla contains exactly 4 PNs, and additional cell types with proposed adhesive and/or sensory functions (Zeng et al., 2019; Johnson et al., 2023b). PNs are also found in species with complex eversible papillae (e.g., *Diplosoma* spp.) and even in those without overtly protrusive papillae, (e.g., *Molgula* spp) (Torrence and Cloney, 1982; Vorontsova et al., 1997). Larval metamorphosis in *Ciona* depends on mechanosensation, as larvae attach to a solid substrate to initiate tail regression and the transition to the post-metamorphic, sessile stage. Mechanical stimulation of the papillae were shown to be sufficient and necessary for triggering metamorphosis, while impairing PN development or function can block metamorphosis (Wakai et al., 2021; Sakamoto et al., 2022; Hoyer et al., 2024).

Like all epidermal neurons in the *Ciona* larva, PNs have apical cilia and axons. Their axons continue to extend posteriorly towards the larval brain during the swimming period, and these potentially late connections coincide with the competence period (Johnson et al., 2023b). Swimming larvae are not immediately competent to initiate tail regression and metamorphosis immediately after hatching, and competence to settle is acquired only after a few hours of swimming (Nakayama-Ishimura et al., 2009), presumably while PN axons are still growing. Unfortunately, the *Ciona* larva connectome was described in a relatively early larval specimen, and these later connections have not been documented at the synaptic level (Ryan et al., 2016). Little else is known about how PNs might regulate tail regression and metamorphosis downstream of mechanical stimulation. It is known that GnRH is important for tail regression, while GABA appears to regulate GnRH release and other processes in metamorphosis, such as body rotation (Hozumi et al., 2020). However, it is unclear where and how these neurotransmitters act, in the absence of PN synaptic connectivity data.

The PNs develop from an anterior neurogenic territory surrounding the central cells of the papillae that shows many similarities to the neurogenic midlines of the tail (Johnson et al., 2023b; Roure et al., 2023). This territory expresses *Ascl.a,* and later on Delta/Notch signaling limits the number of *Pou4*+ cells that will differentiate into PNs (Johnson et al., 2023b). The papilla territory in turn is specified by *Foxc* and *Foxg* orthologs (Horie et al., 2018; Liu and Satou, 2019), which suggests an evolutionary connection to anterior placodes of vertebrate embryos. However, the cells that give rise to PNs appear to downregulate *Foxg,* while sustained *Foxg* expression is associated with the more central papilla cell types, like the Axial Columnar Cells (ACCs) (Johnson et al., 2023b). Knocking out *Pou4* blocks PN differentiation and metamorphosis (Sakamoto et al., 2022; Johnson et al., 2023b). Similarly, using chemogenetics to inhibit PN function also inhibits metamorphosis (Hoyer et al., 2024). Candidate effectors of PN functions have also been knocked down/out, resulting in similar loss of metamorphosis. For instance, morpholino knockdown and TALEN-mediated knockout of a gene encoding the TRP channel family member PKD2 reduced the incidence of mechanically-induced Ca^2+^ transients in PNs and moderately inhibited metamorphosis (Sakamoto et al., 2022). Similarly, CRISPR/Cas9-mediated knockout of *Vamp1/2/3,* encoding synaptic vesicle protein Synaptobrevin, also modestly inhibited metamorphosis (Johnson et al., 2023a). However, the exact mechanotransduction channel in the PNs has yet to be identified. Based on Ca^2+^ imaging, PNs also respond to chemical cues, suggesting they may be polymodal sensory cells (Hoyer et al., 2024). Certain chemicals can promote or inhibit tunicate settlement and metamorphosis, suggesting that the larvae rely on both biotic and abiotic cues for optimal settlement site selection (Durante, 1991; Rae Flores and Faulkes, 2008; Hoyer et al., 2024). Interestingly, the ACCs at the very center of the papillae also respond to mechanical stimuli (Hoyer et al., 2024), which may reflect independent mechanosensory ability, or local communication between PNs and ACCs. In sum, although substantial work is still needed to better understand the development and function of the PNs, they represent a promising model for the study of tunicate mechanosensation thanks to the clear metamorphosis defects associated with their loss or perturbation.

### 2.4 Otolith and antenna cells

Most ascidian larvae have an otolith/statocyst, which is most frequently a single, rounded melanin-containing cell suspended in the lumen of the sensory vesicle (Torrence, 1986; Jiang et al., 2005) (Figures 2A–C). *Ciona* larvae exhibit strong geotactic behavior, preferring to settle on the dark underside of obstacles in the water (Jiang et al., 2005), such as floating docks and ship hulls. A light- and gravity-dependent circuit has been proposed for ensuring such behavior, as larvae will swim up (or position themselves upwards if facing down) when drifting under a shaded area (Bostwick et al., 2020). Key to this behavior are the Antenna Cells, a pair of neurons that make contact with the otolith and presumably detect its displacement in the sensory vesicle via mechanotransduction (Torrence, 1986; Sakurai et al., 2004). However, little is known about these neurons aside from their characterization by the connectome studies, in which they were shown to make extensive synapses onto a handful of relay neurons in the larval brain (Ryan et al., 2016). Therefore, their mechanosensory nature is by far the most speculative and poorly documented out of all the candidate mechanoreceptors of the tunicate larva. A variant of the typical larvae of some colonial species belonging to the taxon Styelidae have only one sensory organ, the photolith, which is thought to function in both gravity and light reception (Sorrentino et al., 2001). In *B. schlosseri*, it consists of a unicellular statocyst, formed by an expanded pigment cup, which receives extensions from six photoreceptor cells (Sorrentino et al., 2001).

## 3 Mechanosensation in adult tunicates

**FIGURE 3:**
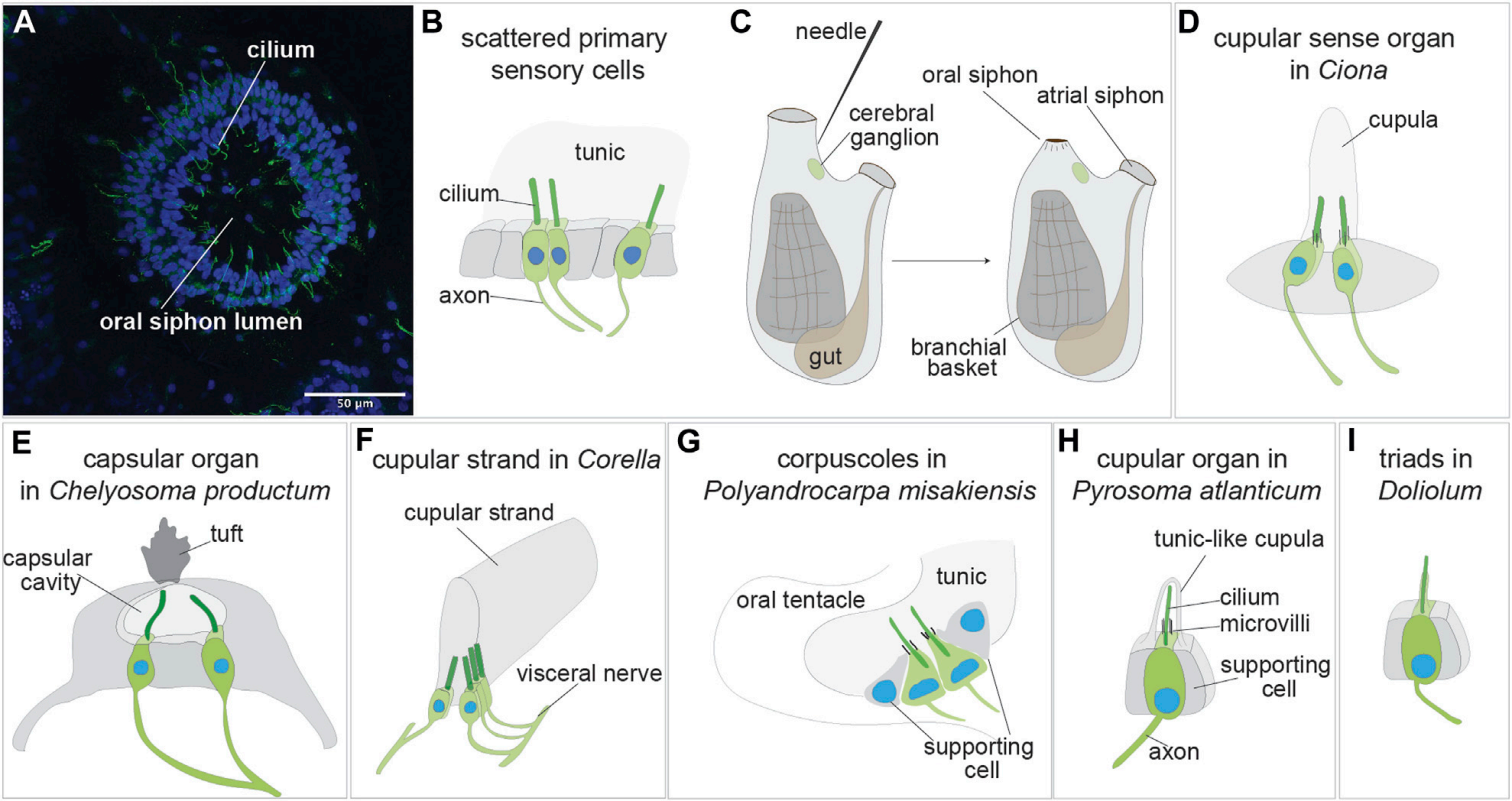
Sensory organs based on primary sensory cells in adult tunicate (see Caicci et al., 2013; Manni and Pennati, 2015; Anselmi et al., 2022). **(A)** Confocal imaging of the primary sensory cells stained with anti-alpha tubulin (green) labelling nerves and Hoechst (blue) in the oral siphon of *B. schlosseri.* **(B)** Scattered primary sensory cells.) **(C)** Illustration of the primary sensory cell stimulation in the “siphon stimulation test”. **(D)** Cupular sense organ in *Ciona*. **(E)** Capsular organ in *Cheliosoma productum*. **(F)** Cupular strand in *Corella*. **(G)** Corpuscles in *Polyandrocarpa misakiensis.* **(H)** Cupular organ in *Pyrosoma atlanticum*. **(I)** Triads in *Doliolum nationalis.*

Mechanoreception in adult tunicates is well developed, relying on both primary sensory cells, which are either scattered, organized in small clusters, or in specific organs (Figure 3), and the secondary sensory cells of the coronal organ (as discussed in Section 3.3). However, information on primary sensory cells and/or organs containing primary sensory cells in adult tunicates is quite limited in comparison to that regarding the ascidian larva. In most cases only morphological data are available with occasional supplementation from the results obtained by neurophysiologists who worked in the field in the 70–90 s of the last century. Even though no developmental data are currently available for these primary sensory cells, developmental data is available for the oral siphons, a very sensitive region where many primary sensory cells are located. Specifically, the oral siphon primordium expresses anterior placode markers Pitx and Dlx, indicating that oral siphon primordia express genes shared with vertebrate placodes (Boorman and Shimeld, 2002; Irvine et al., 2007; Graham and Shimeld, 2013). The comparative morphology of the coronal organ has been deeply analyzed in several tunicate species and some aspects of its development and physiology have been studied in a select few ascidians.

### 3.1 Scattered or clustered primary mechanoreceptor cells in adult tunicates

Isolated primary cells (or small clusters of 2-3 primary sensory cells) have been described in the vicinity of the siphons, the most responsive regions of adult ascidian and thaliaean tunicates [reviewed in (Mackie and Burighel, 2005; Manni and Pennati, 2015)] (Figures 3A, B). These cells are peripheral neurons whose somata are in the monolayered epidermis that both delimits the animal body and descends into the siphons. A long axon extends from the soma base to the central nervous system, whereas a dendrite, represented by a single cilium, projects apically. Since the epidermis is covered by the tunic, these intraepithelial sensory cells are not directly exposed to the external seawater. They react to stimuli through the tunic, which has a different thickness and elasticity depending on species and body region. In the ascidians *Corella inflata* and *Corella eumyota*, each siphon has approximately 8,000 primary sensory neurons that have been revealed by immunohistochemical labeling (Mackie et al., 2006). Physiological tests have shown that these cells are tactile and vibration sensors. In the same siphon region, rounded, axonless cells were also frequently seen, along with cells with very short axons; both have been suggested to be early stages in the formation of the sensory neurons. In the colonial ascidian *B. schlosseri*, the oral siphon primary sensory cells have been analyzed in relation to the blastogenetic cycle and to colony aging (Anselmi et al., 2022) (Figure 3B). The number of these mechanoreceptor cells varies along the cycle, increasing from early-to mid-cycle, before decreasing in late-cycle. This dynamic pattern parallels zooid sensitivity to stimuli, which is greatest when the number of mechanoreceptors is highest. Similarly, both the number of oral siphon primary sensory cells and zooid sensitivity to stimulation are higher in zooids belonging to young colonies than in those belonging to old colonies. In *B. schlosseri*, the ability of these mechanoreceptors to respond to stimuli has been detected using a sensitive behavioral test, the siphon stimulation test (Anselmi et al., 2022) (Figure 3C). This test assesses the ability of the oral siphon to close after stimulation with a waterjet whose pressure is controlled by a microinjector. The waterjet pressure is increased progressively, and the minimum pressure needed to induce a siphon contraction is recorded as a parameter indicating zooid sensitivity.

Primary ciliated sensory neurons, presumed mechanoreceptors, have also been reported in thaliaceans and appendicularians and were described using conventional light microscope staining techniques or Nomarski microscopy [reviewed in (Bone, 1998)]. Unfortunately, detailed morphological observations from transmission electron microscopy (TEM) are not available for these sensory cells. In *Pyrosoma*, those around the inhalant siphon were investigated experimentally, finding that their delicate touch evokes siphon contraction, whereas a stronger stimulation evokes a siphon contraction by branchial ciliary arrest (Mackie and Bone, 1978). In salps, sensory cells with a long cilium were also reported on the mouth lips, sometimes also organized in small groups. However, no detailed or physiological information is available on them (Bone, 1998).

### 3.2 Multicellular mechanoreceptor organs based on primary sensory cells in adult tunicates

A number of multicellular organs with putative mechanoreceptive function have been described morphologically in tunicates, both at light and electron microscopy [reviewed in (Bone, 1998; Mackie and Burighel, 2005; Caicci et al., 2013; Manni and Pennati, 2015)]. In some cases, their mechanoreceptive function has been determined by means of experimental studies; in other cases, it has been inferred on the basis of organ morphology and position. No data are available on their development. Among tunicates, ascidians have been more extensively studied than thaliaceans and larvaceans. The variety of multicellular organs, probably evolved from clusters of simple ciliated mechanoreceptors (Mackie and Singla, 2003), underlines the importance of mechanoreception and its behavioral integration.

The first organs to be described using scanning and transmission electron microscopy were the cupular sense organs (75–100 per individual) located in the atrial mantle epithelium of the adult ascidian *Ciona intestinalis* (Bone and Ryan, 1978) (Figure 3D). They are composed of groups of supporting cells flanking 15–20 ciliated neurons whose sensorial cilia are embedded in a gelatinous cupula, probably produced by the supporting cells. The cupula gives the name to the organs. These organs are able to detect near field vibrations as well as local water movements that displace the cupula and the cilia within it, resulting in electrical responses in the sensory cells. For their overall morphology and physiology, shared with the neuromasts of the lateral line organ and the hair cells of the vertebrate inner ear, the cupular sense organs were suggested by the authors to be evolutionarily linked to the vertebrate mechanosensory organs. From a cellular point of view, however, the cupular sense organs comprise primary sensory cells, whereas the vertebrate counterparts comprise secondary sensory cells, making the hypothesized homology inconsistent.

For many years the cupular sense organs were the only multicellular mechanoreceptor organs known in adult ascidians, until Mackie and Singla described in the atrial wall of the branchial sac of the solitary ascidian *Chelyosoma productum* the capsular organs at light and electron microscopy (Mackie and Singla, 2003) (Figure 3E). In the latter, the sensory cells are grouped in a macula and are characterized by a group of short microvilli surrounding a long cilium projecting into a small cavity (the “capsule”). The capsule cavity is delimited by supporting cells, is filled with a fluid and has an acellular diaphragm spanning an opening in the top. Each sensory cell has an axon reaching the brain via the visceral nerve, the nerve connecting the brain to the visceral organ (branchial sac, gut and heart). By means of electrophysiological recordings and tests aimed to determine their sensitivity, the authors concluded that these organs are vibrational-sensing and are adaptive in detecting the movements of objects in the vicinity.

The same authors described also in the genus *Corella* other organs based on primary sensory cells (Mackie and Singla, 2005). Using immunocytochemical analyses, they found in *C. eumyota* structures resembling the cupular sense organs of *C. intestinalis*, but located on the atrial surface of the branchial sac. Moreover, they recognized in *C. inflata*, using both immunocytochemistry and electron microscopy, a novel sense organ, the cupular strand (Figure 3F) which is a very elongated cupular organ located in the atrial surface of the branchial sac. Axons from the sensory cells enter the cerebral ganglion through the visceral nerve. Neither the cupular sense organs nor the cupular strand have been studied physiologically. However, by analogy with such structures in other metazoans, cupular organs were supposed to be hydrodynamic sensors registering local disturbances or changes in water flow through the atrial cavity.

A similar function was hypothesized for primary sensory cells of the colonial ascidian *Polyandrocarpa misakiensis* (Koyama, 2008). These cells form small corpuscles located in epidermal pockets filled with tunic at the base of the oral and atrial siphons and have been called “oral tentacular sensory cells” and “atrial tentacular sensory cells”, respectively. The sensory cells, both isolated and forming small clusters, have axons joining a nearby nerve located at the base of the siphons (Figure 3G). Their apical apparatus is composed of a long, modified cilium projecting into the tunic, accompanied by a ring of microvilli of equal length. Supporting cells delimit the small cluster of sensory cells or are located between the isolated sensory cells. Some oral tentacular sensory cells are also found associated with neurosecretory cells.

Cupular sense organs have also been described in the thaliacean *Pyrosoma atlanticum* (Pyrosomatida), in a study aimed at describing at electron transmission microscopy the oral sensory structures of this tunicate (Caicci et al., 2013). The organs, previously mentioned by (Fedele, 1923), are scattered on the rounded flaps of the oral siphon and are composed of pyriform sensory cells accompanied by supporting cells (Figure 3H). The sensory cell apical plasmalemma exhibits a long cilium surrounded by 50–60 microvilli and is embedded in a tunic-like cupula secreted by supporting cells. An axon emerges from the sensory cell basal side. The organ function has not been investigated. However, displaying strong morphological resemblance with the ascidian cupular organs, it was supposed they play a similar mechanosensory role, probably in relation to reflex patterns involved in swimming control. Indeed, when the oral siphon is stimulated by touching, or by the collision of large particles or their entry into the gill, the animal responds by arresting ciliary beating and contracting the siphon (Bone and Ryan, 1978). It has been suggested that ascidian and thaliacean cupular organs are the result of evolutionary convergence (Caicci et al., 2013).

In the thaliacean *Doliolum nationalis* (Doliolida), triads of sensory cells, have been described in whole mount preparations (Bone, 1959) and by transmission electron microscopy (Caicci et al., 2013). These are a dozen groups of three sensory cells (supporting cells are not present) regularly arranged around the oral siphon, covered by the tunic. Each sensory cell has an apical long cilium projecting into the tunic and extends an axon from its base (Figure 3I). The triads are stimulated by the deformation of their apical cilia when water flows through the oral siphon as the animal swims (Bone, 1959). The oozooid stage of doliolids displays also an otocyst, but no detailed information is available on its morphology (Bone, 1998).

Apart from a statocyst containing a statolith, located on the left part of the Oikopleuridae brain, whose mechanosensory function has not been studied (Bone, 1998), no other multicellular mechanosensory organs based on primary sensory cells have been found in larvaceans. The ventral organ, a sensory structure below the mouth constituted of about 30 primary ciliated receptors, is considered a chemosensor (Bollner et al., 1986).

### 3.3 Secondary sensory cells in tunicates

**FIGURE 4:**
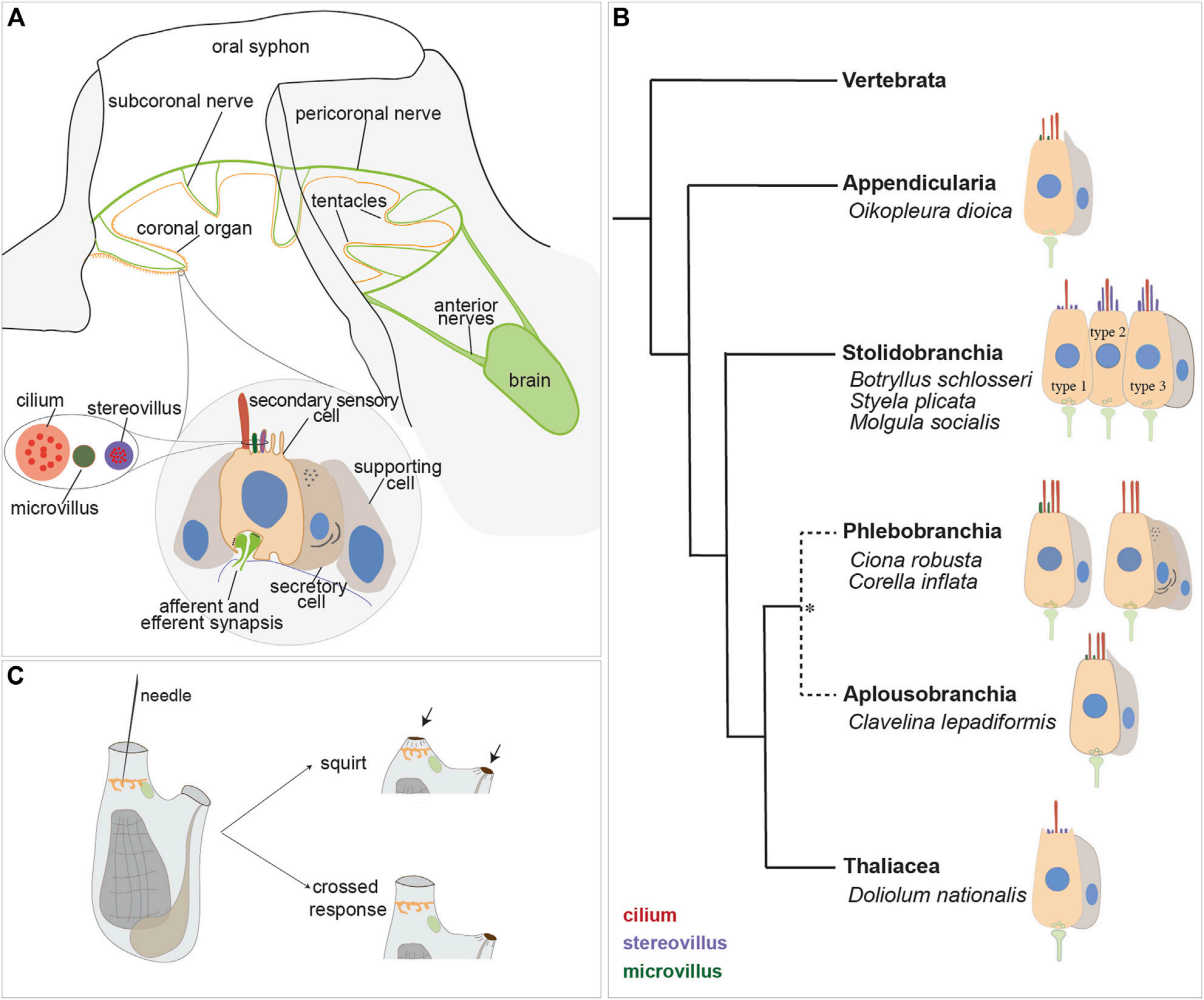
Secondary sensory cells in the adult tunicates. **(A)** Location and main features of the coronal organ in tunicates. The organ is composed of a continuous row of cells on the oral tentacles and the velum (orange). Each sensory cell makes synapses with the subcoronal nerves (two per tentacle, close to the coronal organ) that are branches of the pericoronal nerve (green). The latter is a mixed nerve, connected to the brain through the anterior nerves. Sensory cells (pink) are flanked by supporting cells (grey); in some enterogona species, also secretory cells (violet) can be recognised. Stereovilli are apical, finger-like, long structures, composed of parallel actin filaments connected to the cell cytoskeleton; microvilli are thinner than stereovilli, with less abundant actin microfilaments. **(B)** Comparative schematic illustration showing the coronal organ variability in some representatives of tunicate groups. Stolidobranchia ascidians display the greatest complexity in the sensory apical bundle, which can be composed of microvilli or stereovilli, the latter also graded in length. * The monophyly of Phlebobranchia is disputed [see (DeBiasse et al., 2020)]. **(C)** Responses obtained after a strong (upper) and a light (bottom) stimulation of the coronal cells. The latter response is detected in the “tentacle stimulation test”.

The coronal organ has been found in all examined tunicates except salps (see paragraph 4.1) (Burighel et al., 2011; Caicci et al., 2013; Rigon et al., 2013). Positioned at the outer edge of the velum and the tentacles, at the base of the oral siphon, this organ comprises a continuous row of secondary sensory cells (Figure 4A). These cells are characterized by the presence of numerous stereovilli or microvilli and nonmotile cilia (a single cilium or multiple cilia) composed of 9 + 2 microtubules. The secondary sensory cells form a ring at the base of the oral siphon exposed to incoming water. Indeed, they function as mechanoreceptors involved in detecting variation in water flowing inside the oral siphon and possibly dangerous particles (Mackie et al., 2006).

**FIGURE 5:**
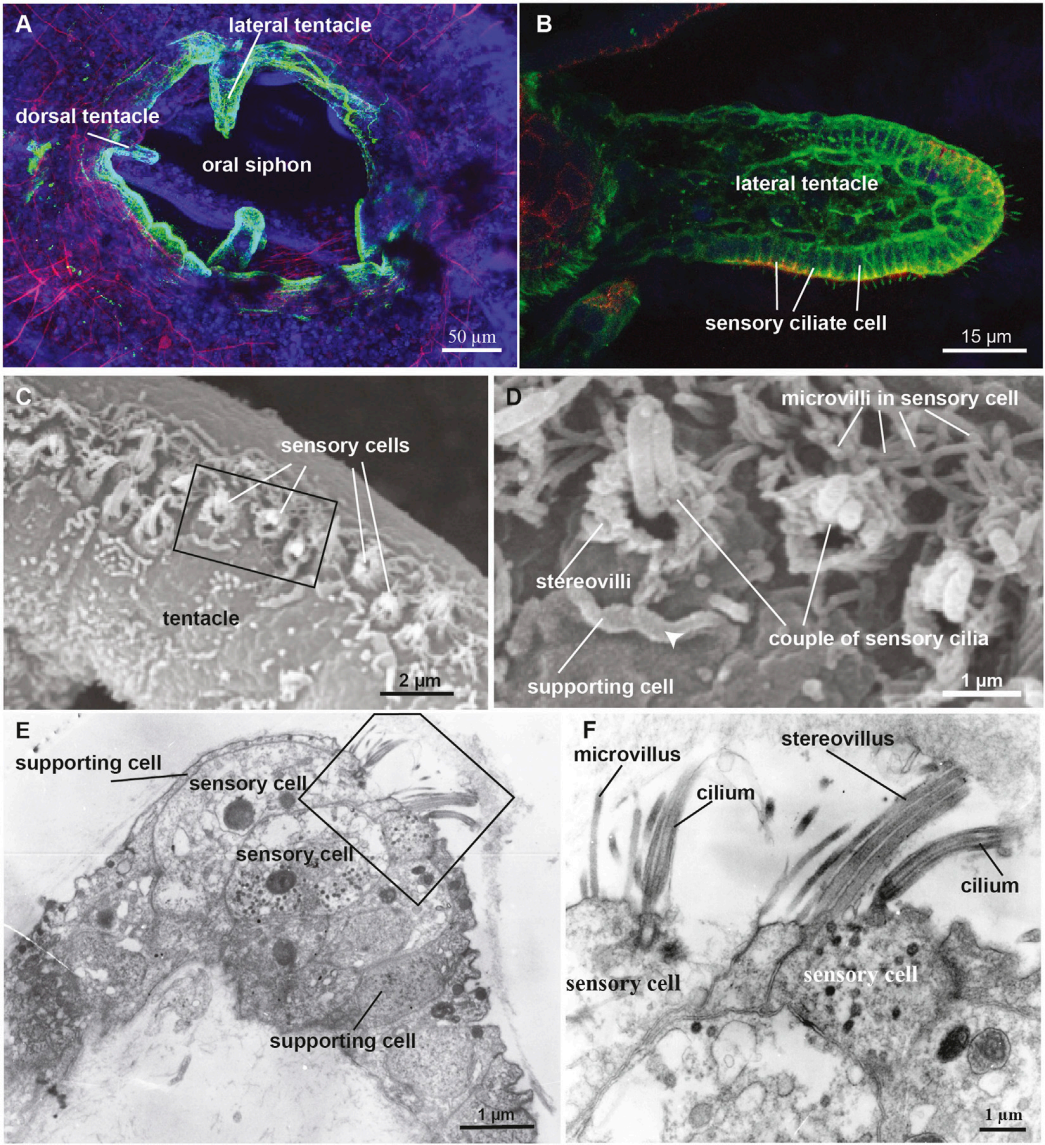
**(A,B)** Confocal pictures of the *B. schlosseri* oral siphon and tentacles stained with anti-alpha tubulin (green) labelling nerves, phalloidin (red) labelling cytoplasmatic actin and dapi (blue) labelling cell nuclei. **(C,D)** Scanning electron microscopy showing the coronal organ of *Molgula socialis*. Squared area in C is enlarged in D. The organ is composed of a row of 1-2 sensory cells (recognisable by their hair bundle) flanked by supporting cells characterized by an apical cytoplasmic crista (arrowhead). Two types of sensory cells can be recognised: with a couple of cilia surrounded by graded stereovilli (type 3), and with a single cilium surrounded by microvilli (type 1). **(E,F)** Transmission electron microscopy showing a transverse section of the coronal organ of *M. socialis*. Squared area in E is enlarged in F to show the different apical bundle structure: two sensory cells at left display microvilli (type 1), whereas the sensory cell at right possesses stereovilli (type 2 or 3).

Both afferent and efferent synapses are found between the base of coronal sensory cells and the peripheral axons of sensory neurons whose cell bodies lie on the brain (Burighel et al., 2003). The innervation pattern of the coronal organ has been studied through immunochemistry (Mackie et al., 2006; Gasparini et al., 2013a; Anselmi et al., 2022), and synaptic connectivity has been established using transmission electron microscopy (Burighel et al., 2003; Manni et al., 2004 ; 2006; Caicci et al., 2010b; Caicci et al., 2013) and *in situ* hybridization (ISH) experiments (Rigon et al., 2018). Each tentacle contains nerve fibers (from the subcoronal nerve) located at the base of the ciliated cells branching from the pericoronal nerve (Figures 4A, 5A, B), a nerve that encircles the oral siphon and originates from the anterior nerve brain. Synaptic contacts have been identified, using TEM, based on the presence of small presynaptic vesicles on one or both sides of the synaptic cleft and the characteristic thickening of the postsynaptic membrane. Frequently, sensory coronal cells make synapses with multiple neurites (Burighel et al., 2011). Glutamate (which mediates afferent hair cell inputs), acetylcholine, GABA and serotonin (which is involved in efferent stimulation to hair cells) are expressed in the coronal organ (Rigon et al., 2018).

The sensory cells are flanked on both sides by supporting cells and, in some species, by secretory cells. Typically, supporting cells extend apically a cytoplasmic crest delimiting the nearby sensory bundle (Figure 4A) and are connected to neighboring cells through tight junctions. There is no gap junction: signal transmission to the central nervous system is solely mediated by neurons located in the brain (Burighel et al., 2011). Secretory cells, when present, face towards the middle of the tentacles and do not form synapses with the nerve that contacts the sensory cells. Their function is not known, however the abundance of ER and the extended Golgi apparatus suggest that they are involved in protein synthesis. Their vicinity to the sensory cells suggests a secretory mechanism activated by the stimulation of the sensory cells (Manni et al., 2006). Sensory cells, supporting cells and, if present, secretory cells are all supported by a basal lamina that consists of a layer of fibers that merge and surround with the nerve fibers.

### 3.4 Variability of coronal sensory cells

The coronal organ exhibits a remarkable degree of diversity among the different tunicate species and even within the same species (Table 1). The diversity of the coronal organ is correlated to the variability of the apical structure and the presence or absence of secretory cells (Figure 4B). In Stolidobranch ascidians, three types of sensory cells have been identified based on the organization of their apical structure: a central cilium surrounded by microvilli (type 1), two long cilia and graded-height stereovilli (type 2), a complete ring of stereovilli surrounding two cilia (type 3). Interestingly, some species like *Styela plicata* can exhibit both type 1 and type 2 sensory cells (Manni et al., 2004). *Molgula socialis* presents a particularly complex condition since displaying all three types of sensory cells with types 2 and 3 predominantly located towards the proximal side of tentacles where they are exposed to inflowing water, while type 1 is located more peripherally (Figures 5C–F). It is worth noting that in Stolidobranch ascidians, extracellular radial filaments connecting the cilia to the surrounding stereovilli have been described (Burighel et al., 2003; Caicci et al., 2010a), even though the precise mechanism of signal transduction is not yet fully understood. In Phlebobranchiata and Aplusobranchiata some species have short microvilli (*Ciona robusta*), while others lack them (*Phallusia mammillata*). Additionally, some species such as *Ascidiella aspersa* and *C. inflata* possess secretory cells. Secretory granules have been found not only in both type 2 and type 3 sensory cells of Pleurogona (Manni et al., 2004; Manni et al., 2006; Caicci et al., 2007) but also in some sensory cells of Enterogona species (Manni et al., 2006).

**TABLE 1:** Table summarizing the principal findings on the tunicate secondary sensory cells.

Taxon	Species	Location	Proposed function	Behavioural test	Hair bundles	Cytoplasm of sensory cell	Radial filament connecting the cilia	Supporting cells	Accessory secretory cells	References
Pleurogona stolidobranchia	Botryllus schlosseri	tentacles of oral siphon	Sensitivity to contact of inflowing particles	Tentacle stimulation test	Single cilium and stereovilli	Accessory centriole	Loose fibrillar matrix generally present among microvilli and cilia	Supporting cells form a wall or crest		Burighel et al. (2003)
	Botrylloides leachi, B. violaceus	tentacles of oral siphon	Sensitivity to contact of inflowing particles		Single cilium and stereovilli	Accessory centriole	Loose fibrillar matrix generally present among microvilli and cilia	Supporting cells form a wall or crest		Burighel et al. (2003), Burighel et al. (2008)
	Molgula socialis	tentacles of oral siphon	Sensitivity to contact of inflowing particles		Three types (type 1,2 and 3). Stereovilli	Electron dense granules, accessory centriole in sensory cells	Extracellular radial filaments connecting the cilium or cilia to the surrounding stereovilli	Supporting cells form a wall or crest		Caicci et al. (2007)
	Pyura stolonifera, P.haustor	tentacles of oral siphon	Sensitivity to contact of inflowing particles		A pair of cilia surrounded by a crescent ring of stereovilli graded in length	Accessory centriole in sensory cells, electron dense granules	Extracellular radial filaments connecting the cilium or cilia to the surrounding stereovilli	Supporting cells form a wall or crest		Caicci et al. (2010a)
	Styela plicata. S. montereyensis, S. gibsi	tentacles of oral siphon	Sensitivity to contact of inflowing particles		A pair of cilia surrounded by a crescent ring of stereovilli graded in length	Accessory centriole in sensory cells, electron dense granules	Extracellular radial filaments connecting the cilium or cilia to the surrounding stereovilli	Supporting cells form a wall or crest		Manni et al. (2004), Caicci et al. (2010a)
	Polyandrocarpa zorritensis	tentacles of oral siphon	Sensitivity to contact of inflowing particles		A pair of cilia surrounded by a crescent ring of stereovilli graded in length	Accessory centriole in sensory cells, electron dense granules	Extracellular radial filaments connecting the cilium or cilia to the surrounding stereovilli	Supporting cells form a wall or crest		Caicci et al. (2010a)
Enterogona aplousobranchia	Clavelina lepadiformis	tentacles of oral siphon	Sensitivity to contact of inflowing particles		More than two cilia of same length that constitute an oriented rows parallel to coronal organ; microvilli			Supporting cells form a wall or crest		Manni et al. (2006)
	Diplosoma listerianum	tentacles of oral siphon	Sensitivity to contact of inflowing particles		More than two cilia of same length that constitute an oriented rows parallel to coronal organ; microvilli			Supporting cells form a wall or crest		Manni et al. (2006)
Enterogona phlebobranchia	Ciona robusta	tentacles of oral siphon	Sensitivity to contact of inflowing particles	Tentacle stimulation test	More than two cilia of same length that constitute an oriented rows parallel to coronal organ; microvilli	Accessory centriole in sensory cells				Mackie et al. (2006), Manni et al. (2006)
	Ascidiella aspersa	tentacles of oral siphon	Sensitivity to contact of inflowing particles		More than two cilia of same length that constitute an oriented rows parallel to coronal organ; microvilli				Accessory secretory cells	Mackie et al. (2006), Manni et al. (2006)
	Phallusia mammillata	tentacles of oral siphon	Sensitivity to contact of inflowing particles		More than two cilia of same length that constitute an oriented rows parallel to coronal organ, no microvilli/stereovilli	Electron dense granules in sensory cells				Mackie et al. (2006), Manni et al. (2006)
	Chelyosoma productum	tentacles of oral siphon	Sensitivity to contact of inflowing particles		More than two cilia of same length that constitute an oriented rows parallel to coronal organ; microvilli				Accessory secretory cells	Mackie et al. (2006), Manni et al. (2006)
	Corella inflata, C. willmeriana	tentacles of oral siphon	Sensitivity to contact of inflowing particles	Tentacle stimulation test	More than two cilia of same length that constitute an oriented rows parallel to coronal organ; no microvilli/stereovilli				Accessory secretory cells	Mackie et al. (2006), Manni et al. (2006)
Appendicularia	Okopleura dioica, O. albicans	lower lip and pharynx	Monitoring particle flow into pharynx		More than two cilia different in lengths and shorter toward the cell edges; microvilli			Supporting cells form a wall or crest		Bone, 1998; Rigon et al. (2013)
Thaliacea	Pyrosoma atlanticum	flaps and a single ventral tentacle	Sensitivity to contact of inflowing particles		Single cilium, stereovilli					Caicci et al. (2013)
	Doliolum nationalis	flaps	Sensitivity to contact of inflowing particles		Single cilium, stereovilli					Caicci et al. (2013)
Salpe	Thalia democratica	absent								Rigon et al. (2013)

In larvaceans the ultrastructure of the coronal organ was studied in two species of *Oikopleura* (Rigon et al., 2013). These animals have a single type of secondary sensory cells with numerous cilia of the same length, microvilli in multiple lines. They are flanked by non-ciliated supporting cells forming a crest alongside the coronal organ. A recent study on the mesopelagic giant appendicularian *Bathchordeaus stygius* has revealed the presence of three pairs of oral sensory organs within the mouth cavity, hypothesized to be homologous to the coronal organ. Each of these organs is composed of sensory cells with an apical cilium, innervated by brain nerves and surrounded by non-ciliated epidermal cells that nearly cover the organ (Le et al., 2023). In addition to the coronal organ, appendicularians possess the so-called Langerhans cells, which are secondary mechanoreceptors located in the posterior of the “trunk” epidermis and connected with afferent neurites through gap junctions. When stimulated, Langerhans cells trigger the escape response of the animal (Bone and Ryan, 1978).

In thaliaceans, the coronal organ has been studied in *Pyrosoma atanticum* and *D. nationalis* (Caicci et al., 2013). These animals have a single type of secondary sensory cell possessing a cilium accompanied by microvilli. Instead of tentacles, at the base of the oral siphon thaliaceans have flaps (*D. nationalis*), or single ventral tentacles with dozens of flaps (*P. atlanticum*). Notably, the coronal organ is absent in *Thalia democratica*, a salp. This absence is likely a derived condition evolved in parallel with the different feeding system adopted by this group of animals. Indeed a cladistic analysis, performed using 19 morphological characters in 16 tunicates species, and a cephalochordate and three vertebrate species as outgroups, revealed that the putative ancestral coronal cell in tunicates was a simple monociliated cell, that successively differentiated into the current variety of oral mechanoreceptors in the various tunicate lineages. The evolutionary changes in sensory cells may correspond to different feeding strategies (Rigon et al., 2013).

### 3.5 Physiology of the coronal organ

Studies aimed to elucidate the function of the coronal organ have primarily focused on two species: the solitary ascidian *C. inflata* (Mackie et al., 2006) and more recently the colonial ascidian *B. schlosseri* (Manni et al., 2018; Anselmi et al., 2022; Thompson et al., 2022). Behavioral experiments aimed to manipulate water flow patterns and observe siphon closure responses demonstrated that the secondary sensory cells are mechanoreceptors (Mackie et al., 2006). In *C. inflata,* a pioneering study showed that stimulating the oral tentacles with a glass needle caused the atrial siphon to contract to less than half its resting diameter, with no change in the diameter of the oral siphon. This response was named “crossed response” (Figure 4C). Depending on the stimulus strength and duration, the degree of atrial siphon closure during the crossed response varied. While gentle stimulation of the inner surfaces of the siphon or oral tentacles elicited varying degrees of the crossed response, stronger stimulation induced “squirts”, characterized by a robust, synchronous contraction of both siphons and adjacent regions of the body wall (Figure 4C). This was accompanied by arrest of the cilia activity in the branchial stigmata responsible for creating the water current. Notably, a single stimulation could evoke not just one but a series of contractions suggesting coordination through a pacemaker (Mackie et al., 2006). These responses were lost after tentacle amputation. Electrophysiological recordings on the oral siphon were conducted to measure the electrical activity of the secondary sensory cells when exposed to specific stimuli in order to understand how sensory cells are activated and transmit signals. The results confirmed that crossed responses and squirts are centrally mediated reflexes but local conduction pathways also exist and persist after brain removal (Mackie et al., 2006).

Further insights have emerged from a different type of behavioral experiment, the tentacle stimulation test, conducted in *B. schlosseri* to assess animal performance under different conditions (Manni et al., 2018; Anselmi et al., 2022). This test aims to record the minimum pressure applied to the tentacle required to trigger the crossed reflex. Controlled and quantifiable pressure was applied through a water jet flow directly to the tentacles. Results showed that stage of adult individuals, the age of the colonies, and their overall condition (*e.g.*, exposure to drug) significatively influence the zooids performance. Specifically, a higher threshold for response is observed in case of lower numbers of brain neurons, as in old colonies and zooids approaching their resorption, or in case of coronal organ impairment following drug treatment. In this regard, is it important to mention that the coronal sensory cells, like vertebrate hair cells, are damaged by gentamicin (an ototoxic drug) treatment resulting in a loss of coronal sensory cell continuity along the organ (Manni et al., 2018). This leads to a significant decrease in the percentage of responsive zooids to the tentacle stimulation test compared to the same colonies before treatment. Interestingly, fenofibrate has been found to have a strong protective effect on coronal sensory cells against the gentamicin-induced toxicity, similar to what occurs in vertebrate hair cells (Park et al., 2017; Manni et al., 2018).

Additionally, experiments involving stimulation of the oral siphon with ultrasound were conducted on three solitary ascidians. These experiments revealed that the coronal organ plays a role in perceiving ultrasounds, exhibiting a frequency-dependent behavioral response. Higher sensitivity was observed at the highest frequency tested (Varello et al., 2023).

### 3.6 Secondary sensory cell development

**FIGURE 6:**
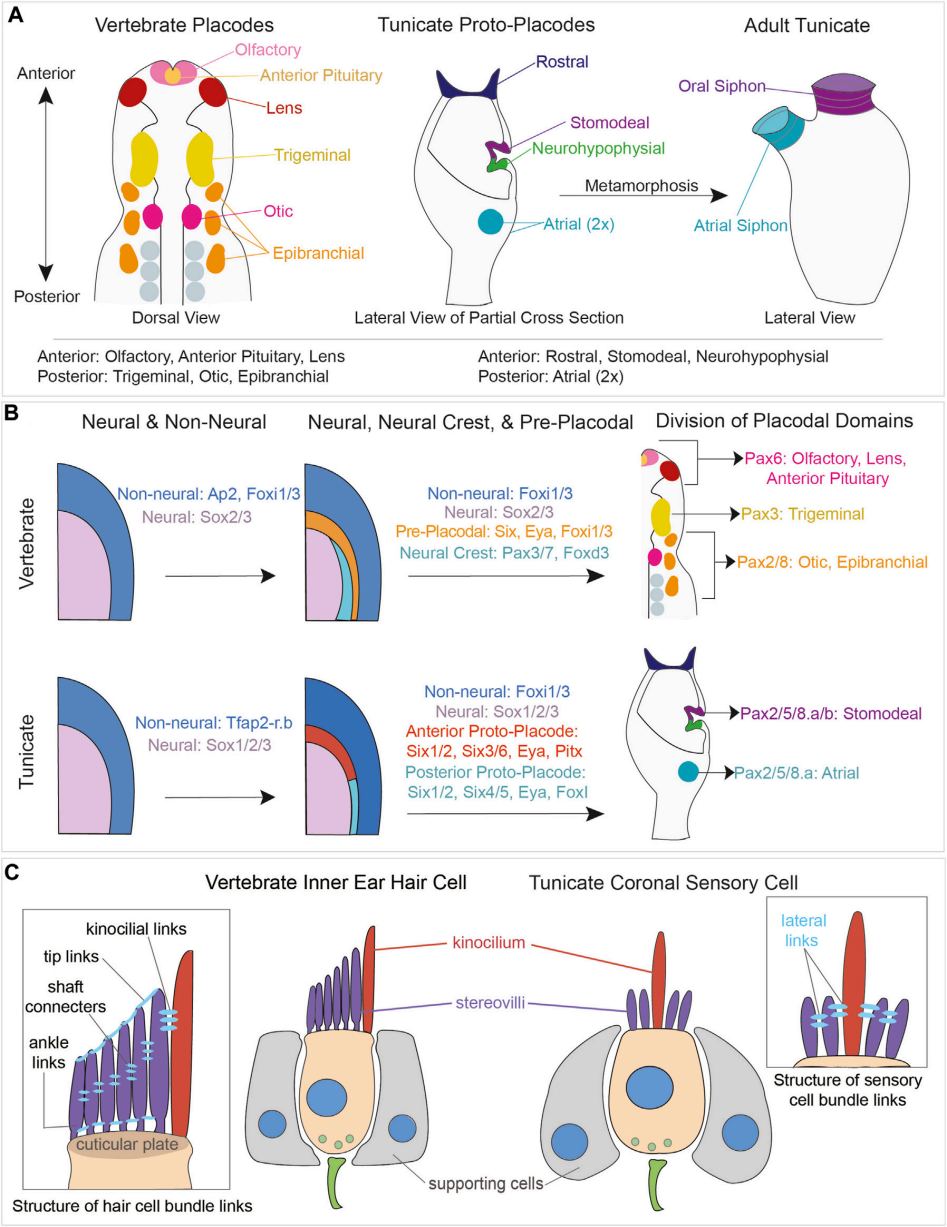
Comparison of vertebrate placodal and tunicate proto-placodal development and vertebrate hair cell and tunicate coronal sensory cell structures. **(A)** Schematic of vertebrate placodes compared to tunicate proto-placodes. The anterior placodes include the olfactory, anterior pituitary, and lens placodes. The posterior placodes include the trigeminal, epibranchial, and otic placodes. Tunicates have three anterior proto-placodes:, the rostral, stomodeal, and neurohypophysial placodes. Tunicates have two posterior atrial proto-placodes. Following metamorphosis, the stomodeal proto-placode will give rise to the oral siphon and the atrial proto-placodes will fuse to form the atrial siphon. **(B)** Conservation of genes expressed during vertebrate placodal and tunicate proto-placodal development. Several key genes involved in placode development appear to be conserved. **(C)** Comparison of hair cells from vertebrates and coronal sensory cells from tunicates. Vertebrate hHair cells (tan) are flanked by supporting cells (gray). Sensory cells possess kinocilium (red) and stereovili (purple) that are connected together by different links.

In tunicates, the coronal organ develops during embryogenesis from a thickened ectodermal epithelium known as the “anterior proto-placode”. This tissue eventually gives rise to the oral siphon, tentacles and velum (Manni et al., 2004; Gasparini et al., 2013a; Manni et al., 2018). Importantly, the anterior proto-placode expresses homologues of some placodal genes (Patthey et al., 2014), (Figure 6).

The development of the coronal organ has been studied using TEM in *C. robusta* and *B. schlosseri* (Manni et al., 2004; Gasparini et al., 2013a). In *Ciona,* coronal cells become morphologically recognizable during the early juvenile stage when they appear as cells with short cilia and occasional microvilli. Over time, these cells progressively develop hair cell-like features, including microvilli containing actin and myosin, and associated with supporting cells. Importantly, the coronal organ continues to grow throughout the entire lifespan of the animal, parallel to the growth of the tentacles. Immunohistochemistry has revealed higher mitotic activity in the coronal organ of adults, with proliferating coronal sensory cells and supporting cells detected using Histone H3 antibody (Gasparini et al., 2013a). ISH has shown that orthologs of genes such as *Atoh1*, *Notch*, *Delta-like*, *HES,* and *Musashi* are expressed during the development of the coronal organ (Rigon et al., 2018) mirroring gene expression patterns seen in vertebrate neural and hair cell differentiation (Fritzsch and Elliott, 2017).

The development of the coronal organ during asexual reproduction has been investigated i*n B. schlosseri* (Manni et al., 2018). The coronal cells undergo cyclical development on a weekly basis becoming first recognizable in the buds during mid-cycle due to the differentiation of their apical bundle and basal synapses. Their definitive configuration is reached when their parents are in late-cycle which coincides with the degeneration of both the parent brain and sensory system.

## 4 Evolutionary relationships between vertebrate and invertebrate mechanoreceptor cells

In addition to primary mechanosensitive sensory neurons, vertebrates possess specialized secondary mechanosensory cells, including the hair cells of the inner ear and lateral line and Merkel cells of the skin. Vertebrate hair cells share several developmental, morphological, and functional similarities with tunicate primary and secondary mechanoreceptor cells. These similarities and differences are discussed below.

### 4.1 The development of vertebrate hair cells from cranial placodes

Towards the end of gastrulation, the vertebrate neural plate arises from the most dorsal population of embryonic ectoderm in response to a variety of organizing signals (Grocott et al., 2012; Groves and LaBonne, 2014; Thawani and Groves, 2020). The border between the developing neural plate and the future epidermis gives rise to two distinct multipotential lineages - neural crest cells and cranial placodes. The cranial placodes are ectodermal thickenings that give rise to (1) cranial sensory neurons of the trigeminal ganglia, (2) cranial sensory neurons of the epibranchial ganglia, (3) the otic placode which will form the entire inner ear including hair cells and sensory neurons, (4) the olfactory epithelium, (5) the lens of the eye, and (6) the anterior pituitary gland (Singh and Groves, 2016; Streit, 2018); (Figure 6A). In aquatic vertebrates, lateral line placodes will give rise to the hair cell-bearing neuromasts located along the head and trunk of the animal. The anterior and posterior lateral line neuromasts of teleosts, named for the direction in which their cells migrate, arise from a lateral line placode in the vicinity of the otic placode. Some aquatic species, like axolotls and paddlefish, have ampullary organs containing specialized electroreceptor cells that also arise from a lateral line placode (Baker et al., 2013; Modrell et al., 2017).

Cells destined to give rise to cranial placodes first express members of the AP2a and FoxI families, which distinguish non-neural ectoderm from the adjacent neural ectoderm (Ohyama and Groves, 2004; Grocott et al., 2012; Khatri et al., 2014; Pla and Monsoro-Burq, 2018); (Figure 6B). Some cells within this region then express both Six homeobox transcription factors and Eya co-regulators in what has been termed the pre-placodal domain (Streit, 2004; 2007; Sato et al., 2010). *Six1* is expressed throughout the pre-placodal domain, posterior placodes like the otic placode express *Eya1* and *Six4*, and anterior placodes like the olfactory placode express *Eya2* and *Six3* (Streit, 2004; 2018; Sato et al., 2010). Locally acting signals then divide this pre-placodal region into distinct placodal territories along its anterior-posterior axis. Members of the Pax gene family play an important role in this process: *Pax6*-expressing progenitors give rise to the olfactory and lens placodes, *Pax3*-expressing progenitors give rise to the trigeminal placode, and *Pax2/8*-expressing progenitors give rise to the otic placode and the epibranchial placodes (Baker and Bronner-Fraser, 2001); (Figure 6B).

Once the otic placode has formed, the tissue transforms by invagination into the otic vesicle or otocyst. The otocyst co-opts dorso-ventral and anterior-posterior signals used to pattern the central nervous system (Groves and Fekete, 2012; Wu and Kelley, 2012) to form a series of prosensory patches expressing the *Sox2* transcription factor (Raft and Groves, 2015). These prosensory patches will give rise to the auditory and vestibular epithelium containing hair cells and supporting cells. The surrounding non-sensory epithelium of the ear expresses the transcription factor *Lmx1a* and will give rise to structures such as the semicircular canals of the vestibular system (Gu et al., 2016; Żak and Daudet, 2021). Mutual antagonism between *Sox2* and *Lmx1a*, driven in part by Notch pathway signaling, leads to the correct positioning and distribution of the prosensory patches (Mann et al., 2017). Hair cells and supporting cells develop from each sensory patch through a process of Notch-mediated lateral inhibition. Differentiating hair cells express Notch ligands to suppress a hair cell fate in neighboring cells, which then differentiate as supporting cells (Basch et al., 2016; Brown and Groves, 2020). Below, we discuss evidence for conservation of these developmental events in the formation of coronal sensory cells in tunicates.

### 4.2 What elements of vertebrate placode development are shared in tunicates?

The presence of thickened, placode-like structures in tunicates was first suggested by a study of the neurohypophysial duct, which generates the neural gland rudiment and migratory cells that contribute to the cerebral ganglion (Manni et al., 2005; 2001; 1999). The discovery of secondary hair cell-like cells in the coronal organs of *Botryllus* and *Ciona* (Burighel et al., 2003; Manni et al., 2005) suggested they may also derive from placodal structures. Subsequent analyses identified four thickened placodal-like structures or “proto-placodes” in tunicate larvae: (1) the rostral proto-placode which will form the larval adhesive organ and its associated sensory neurons, and the adult stolon (Section 2.4), (2) the stomodeal proto-placode that will give rise to the oral siphon including the coronal organ, (3) the afore-mentioned neurohypophysial proto-placode, and (4) the atrial proto-placode which will form the atrial siphon and atrial chamber wall, containing primary mechanosensory cells of the cupular organs (Manni et al., 2004; Gasparini et al., 2013b); (Figure 6A). Subsequent studies analyzed the expression of vertebrate placodal patterning gene orthologues in the developing tunicate proto-placodes at the embryonic, larval, and juvenile stages of *Ciona* and *Botryllus* (Mazet and Shimeld, 2005; Gasparini et al., 2013b). The *Ciona* orthologs of *AP2a* and *Sox2/3*, *Tfap2-r.b* and *Sox1/2/3*, respectively promoted epidermal and neural fate of ectoderm cells (Imai et al., 2017). Members of the Six and Eya families were expressed in both the anterior (stomodeal/neurohypophysial) and posterior (atrial) proto-placodes, with *Six1/2* marking both structure placodes and *Six3/6* being confined to the anterior proto-placode. Tunicate *FoxI* orthologues were expressed in the posterior atrial proto-placode (Mazet and Shimeld, 2005; Gasparini et al., 2013b); (Figure 6B).

Based on these studies of two evolutionarily distant tunicate species, it has been proposed that the tunicate anterior proto-placodes resemble the vertebrate olfactory/lens/hypophyseal placodes, and the tunicate posterior proto-placode resembles the vertebrate otic/epibranchial/lateral line placodes (Gasparini et al., 2013b). However, in vertebrates only the *posterior* otic and lateral line placodes produce hair cells (Groves and LaBonne, 2014; Piotrowski and Baker, 2014), whereas in tunicates coronal sensory cells that most closely resemble vertebrate hair cells are derived from the *anterior* proto-placode. Only the primary mechanosensory cells of the cupular organs are derived from the posterior placode (Gasparini et al., 2013b). Since Six and Eya genes are initially expressed throughout the vertebrate pre-placodal domain (Streit, 2007; Schlosser, 2014), it is likely that additional transcription factor combinations are required to divide this domain more precisely into individual placodes, or that individual placodes are specified at different times. In this regard, it is interesting to note that vertebrate Foxi1/3 genes are initially expressed throughout the pre-placodal domain, at a time when all parts of the pre-placodal domain are competent to generate the otic placode (Solomon et al., 2003a; 2003b; Ohyama and Groves, 2004; Birol et al., 2016) and are gradually downregulated in an anterior-posterior direction (Khatri et al., 2014; Birol et al., 2016). However, Foxi1/3 appear to be required for the development of only the posterior otic, epibranchial and lateral line placodes in vertebrates (Solomon et al., 2003a; Hans et al., 2004; Birol et al., 2016). Rigon and others (Rigon et al., 2018) speculated that the common ancestor of vertebrates and tunicates may have generated mechanosensory cells from both anterior and posterior placode-like structures, with the ability to generate such cells being lost from the anterior placodes in vertebrates and from the posterior ptoto-placodes in tunicates. One possible explanation for this difference is that the evolution of the tunic covering the exterior surface of the animal placed constraints on where mechanosensory cells could function, restricting them to oral structures on the interior of the body that do not have a tunic covering (Manni et al., 2006).

It should be stressed that the putative homology between a Foxi1/3 and Pax2/8-expressing atrial primordium and the vertebrate “otic-epibranchial progenitor domain” is still far from settled (Graham and Shimeld, 2013; Patthey et al., 2014). A n alternative explanation for this paradox is that the posterior region of the tunicate larval head that expresses Six and FoxI genes does not give rise to sensory structures at all. Vertebrate Six and Foxi1/3 genes are also expressed in the developing pharyngeal arch region of vertebrates at a slightly later stage than their expression in the pre-placodal domain (Ohyama and Groves, 2004; Khatri and Groves, 2013; Edlund et al., 2014; Birol et al., 2016; Ankamreddy et al., 2023), and they are required for correct formation of the pharyngeal arch structures (Solomon et al., 2003a; Nissen et al., 2003; Edlund et al., 2014). Interestingly, FoxI and Six orthologues are also expressed in the branchial fissures (stigmata) of the tunicate atrium where peribranchial and branchial epithelium contact each other and fuse (Gasparini et al., 2013b). This expression pattern is reminiscent of the requirement for Foxi1/3 in the vertebrate pharyngeal pouches and clefts that form by fusion of pharyngeal ectoderm and endoderm (Edlund et al., 2014; Hasten and Morrow, 2019). Thus, while Six and FoxI genes mark the posterior atrial proto-placode in both *Ciona* and *Botryllus*, it is possible that these genes are acting to regulate formation of the atrium itself, rather than the cupular mechanosensory cells in the atrial walls. It may be possible to test the function of tunicate Six and FoxI orthologues by CRISPR-based loss-of-function studies to determine if they are necessary for the formation of primary cupular mechanosensory cells in the atrium or only for the formation of the branchial fissures.

As discussed above, vertebrate placodes acquire their unique identity by expression of different Pax family genes. *Ciona* has six Pax family genes, and, although several are expressed in regions of the larval central nervous system, most do not appear to be expressed in any of the proposed proto-placode structures identified in tunicate larvae (Mazet et al., 2003; Imai et al., 2004; Hudson and Yasuo, 2005). Of the Pax genes in *Ciona*, *Pax2/5/8.a* is expressed in the larval atria and stomodeum cavities, and *Pax2/5/8.b* is expressed weakly in the invaginating stomodeum (Mazet et al., 2003; Mazet and Shimeld, 2005). It remains an open question whether any other Pax genes play a role in the formation or patterning of other placode-like structures in tunicates. For example, *Pax6* gene family members are well known to regulate eye development across most animal phyla (Kozmik, 2008). In vertebrates, *Pax6* has also been co-opted to regulate the formation of the lens through its expression in the lens placode (Cvekl and Ashery-Padan, 2014). Ciona *Pax6* is expressed in parts of the brain associated with the photoreceptive ocellus and has the three “lens” cells lying above the ocellus, although they are not believed to be homologous to vertebrate lens cells. These “lens” cells do not express *Pax6*, nor do they express beta-crystallin (Shimeld et al., 2005). Moreover, vertebrate *Pax6* genes have a lens-specific enhancer that is not present in tunicates (Irvine et al., 2008), suggesting that the co-option of *Pax6* to regulate development of a lens structure occurred after vertebrates and tunicates diverged. Clarification of the role of other Pax genes in tunicate placode derivatives will require more sensitive tools to localize their transcripts, such as single cell RNA-seq and *in situ* hybridization, and to test gene function using loss of function approaches such as CRISPR-Cas9.

### 4.3 What elements of vertebrate hair cell development are shared in tunicates?

As described above, vertebrate inner ear and lateral line hair cells develop from patches of prosensory tissue marked by *Sox2*, a member of the SoxB transcription family (Neves et al., 2013). SoxC family members, such as *Sox4* and *Sox11* then act within these patches to provide competence for differentiation of hair cells and supporting cells (Gnedeva and Hudspeth, 2015; Wang et al., 2023). Cells within this expression domain upregulate the proneural transcription factor *Atoh1*, which initially marks the progenitors of both hair cells and supporting cells (Yang et al., 2010; Li et al., 2022). ATOH1, which is both necessary and sufficient for hair cell fate, is quickly restricted to differentiating hair cells through Notch-mediated lateral inhibition (Jarman and Groves, 2013; Cai and Groves, 2015). ATOH1 regulates other transcription factors, such as GFI1 and POU4F3, to establish a hair cell gene regulatory network (Iyer and Groves, 2021; Iyer and Groves, 2021). GFI1 can act with ATOH1 to positively promote the expression of hair cell genes and can also act alone to inhibit expression of neuronal genes (Jen et al., 2022; Jen et al., 2022). Since ATOH1 is also responsible for the differentiation of neurons in the cerebellum, brainstem, and spinal cord (Ben-Arie et al., 1997; Lai et al., 2011; Wu et al., 2023), it is possible that secondary mechanosensory receptor cells co-opted *Gfi1* to repress neuronal gene networks during evolution. POU4F3 also promotes hair cell differentiation by acting as a feed-forward pioneer factor: it is first induced by ATOH1 and then binds to many other ATOH1 target genes to make them transcriptionally accessible (Yu et al., 2021). The combined action of ATOH1, GFI1, and POU4F3 promotes a hair cell fate, and these transcription factors are also capable of reprogramming embryonic stem cells, fibroblasts, or non-sensory cells of the ear to a hair cell fate (Costa et al., 2015; Menendez et al., 2020; Iyer et al., 2022).

Several lines of evidence suggest that these three transcription factors have an evolutionarily conserved role in the differentiation of mechanosensory cells. *Drosophila* orthologues of *Atoh1*, *Gfi1,* and *Pou4f*3 (*atonal*, *senseless* and *acj6* respectively) are expressed in developing chordotonal organs that have mechanosensory functions in proprioception, hearing, and balance (Jarman et al., 1993 ; 1995; Nolo et al., 2000; Lee and Salvaterra, 2002). Significantly, *Atoh1* and *Gfi1* can functionally replace *atonal* and *senseless* in *Drosophila,* and *atonal* can functionally replace *Atoh1* in mice (Ben-Arie et al., 2000; Wang et al., 2002; Acar et al., 2006). Orthologues of *Atoh1*, *Gfi1* and *Pou4f3* have also been identified in *Caenorhabditis elegans* (*Atonh1/lin-32, Gfi1/pag-3,* and *Pou4/unc-86*) and are necessary for the formation of AVM/PVM mechanosensory neurons (Baumeister et al., 1996; Zhao et al., 2020). A Class IV POU gene orthologue has also been shown to be necessary for the development of cnidarian (sea anemone) hair cell-like cells, although it is not known whether *atonal-*like factors regulate this gene in sea anemones (Ozment et al., 2021). Finally, *Atoh1*, *Gfi1,* and *Pou4f3* are all expressed in the touch-sensitive Merkel cells of the skin (Lumpkin et al., 2003; Haeberle et al., 2004; Yu et al., 2021), and *Atoh1* and *Pou4f3* are both necessary for the differentiation of these cells (Maricich et al., 2009; Yu et al., 2021). Significantly, the inductive and pioneer feed-forward relationship between *Atoh1* and *Pou4f3* seen in hair cells is also conserved in Merkel cells, even though they regulate overlapping but distinct sets of genes in these two different mechanosensory cell types (Yu et al., 2021).

It is interesting to speculate on what gene networks are regulated by *Atoh1*, *Gfi1,* and *Pou4f3* orthologues in the different kinds of mechanosensory cells described in the previous paragraph. In a very simplified view, a mechanosensory cell requires (1) membrane specializations to detect mechanical force (such as vertebrate stereovilli or arthropod ciliated dendrites); (2) membrane components to develop a receptor or axon potential; (3) a synaptic apparatus to allow propagation of the mechanosensory stimulus to downstream neurons. These functional modules are created by gene networks expressed during development and then homeostasis. When comparing the molecular identity of cell types, it is important to functionally contextualize homologous genes across species. For example, gene networks regulating synaptic specializations are likely to be more highly conserved between different mechanosensory cells compared to networks regulating the more varied types of force-detecting machinery in these different cell types. Supporting this idea, a recent study comparing vertebrate hair cells and Merkel cells found that genes directly regulated by ATOH1 and POU4F3 in both cell types tended to be associated with synapses, cation channels and potassium channels (Yu et al., 2021). While some modules expressed by an ancestral mechanosensitive cell type may have been conserved, it is also possible that comparable modules were convergently evolved. At present, we have little information on how the development of tunicate mechanosensory cells is regulated. There is currently no evidence that the coronal sensory cellscells and supporting cells of the coronal organ derive from a *SoxB/SoxC*-expressing domain analogous to the prosensory patches of vertebrates. *Atonal* and *Pou4* orthologues are present in *Ciona* and are expressed in larval ciliated epidermal sensory neurons; in these cells *CiAtonal* has been reported to be epistatic to *CiPouf4* (Tang et al., 2013). The coronal organs of *Ciona* express an *Atonal* orthologue, as well as members of the Notch pathway (Rigon et al., 2018), but these genes have yet to be definitively localized to coronal sensory cells or supporting cells. With the advent of molecular techniques such as single cell RNA-seq and CUT&RUN/CUT&Tag, it will become feasible to identify gene networks expressed in mechanosensory cells of different species and to identify the direct targets of transcription factors such as ATOH1, GFI1, and POU4F3 within these networks.

As discussed above, the development of tunicate coronal organ sensory cells from the anterior, stomodeal proto-placode differs from that of hair cells of the vertebrate inner ear and lateral line, which develop from posterior (otic and lateral line) placodes. Although evolutionary scenarios have been proposed to account for this difference (Rigon et al., 2018), the limited data on expression of downstream placodal and prosensory genes in tunicates makes it hard to define the pathways by which coronal sensory cells form in the developing coronal organ. Indeed, it is possible that expression of *Atonal* orthologues in the coronal organ epithelium is sufficient to generate coronal sensory cells and supporting cells without the need to pass through a pre-placodal, placodal or prosensory state. In support of this idea, the chordotonal organs of *Drosophila* are generated by upregulation of *atonal* in embryonic ectoderm to form sensory organ precursors, and over-expression of *atonal* or *Atoh1* is sufficient to generate ectopic chordotonal organs in embryonic ectoderm (Jarman et al., 1993; 1995; Ben-Arie et al., 2000). Merkel cells of the vertebrate skin are generated directly from keratin-expressing epidermis without passing through a *Sox2*+ prosensory phase; here SOX2 appears to control the maturation of Merkel cells, rather than their specification (Lesko et al., 2013; Perdigoto et al., 2014). Finally, activation of *Atoh1*, *Gfi1,* and *Pou4f3* in primary mouse fibroblasts is sufficient to induce many aspects of the hair cell gene regulatory network without prior activation of *SoxB* or *SoxC* factors (Menendez et al., 2020). Localization of *Atoh1* and *Sox2/SoxB* orthologues in developing and mature coronal organ tentacles may help to address some of these questions and to more accurately identify the stages of differentiation of these cells.

### 4.4 What elements of vertebrate hair cell regeneration are shared in tunicates?

Many vertebrate inner ear and lateral line hair cells undergo gradual turnover and replacement in mature animals, and non-mammalian vertebrates can also robustly regenerate new hair cells after the endogenous hair cells are killed (Stone and Cotanche, 2007; Kniss et al., 2016). In non-mammalian vertebrates, new hair cells are generated by the upregulation of *Atoh1* in supporting cells, which then trans-differentiate to a hair cell fate (Stone and Cotanche, 2007). This can occur with or without supporting cell division, but ultimately leads to full replacement of hair cells and functional recovery. The one exception to this is mammals, where the cochlea is unable to regenerate new hair cells after the onset of hearing and the vestibular system is capable of only a modest amount of turnover and regeneration (Groves, 2010; Bucks et al., 2017). Given the ability of other vertebrates to regenerate hair cells, it is possible that with the ancestral form that gave rise to hair cells also had the capacity to regenerate. If tunicate coronal sensory cells and vertebrate hair cells have a shared evolutionary origin, do tunicate coronal sensory cells regenerate? As adult tunicates mature, the tentacles of the oral siphon continue to grow, implying that there must be some post-metamorphic mechanism to generate new coronal sensory cells and supporting cells. Transmission electron microscopy has revealed rare instances of apparently dividing sensory cells in *Pyura haustor* (Caicci et al., 2007), and analysis of mitosis by PH3 staining in the coronal organs of adult and juvenile *C. intestinalis* indicates that both supporting cells and coronal sensory cells are capable of creating new coronal sensory cells (Gasparini et al., 2013a). Recent work suggests that exposure of tunicate coronal sensory cells to the ototoxic aminoglycoside gentamicin leads to an apparent loss of some sensory cells from the tentacles and impairs responsiveness of the coronal organ tentacles to touch (Manni et al., 2018). However, it is not known whether coronal sensory cells can be regenerated after such damage, nor whether any new coronal sensory cells are generated by neighboring supporting cells. Further studies are required to explore the potential for tunicate coronal sensory cell regeneration, and whether genes associated with hair cell regeneration in vertebrates like *Atoh1* play a role in this process.

### 4.5 A consideration of mechanotransduction in vertebrate hair sells and tunicate coronal sensory cells

Vertebrate hair cells are exquisitely sensitive mechanoreceptors; the human ear can detect sounds that vibrate the eardrum by one picometer. Hair cells have a hair bundle protruding from their apical surface consisting of a graded, stair-case-like array of long modified microvilli termed stereocilia or stereovilli (Vélez-Ortega and Frolenkov, 2019); (Figure 6C). Vertebrate hair cells develop with a single true cilium or kinocilium that migrates to an eccentric position on one side of the apical surface of the hair cell as the hair bundle develops (Frolenkov et al., 2004). The kinocilium persists in most mature vertebrate hair cells but degenerates in mammalian cochlear hair cells prior to the onset of hearing (Wang and Zhou, 2021). The apical tips of all but the longest stereovilli are joined to the next tallest stereovillus by a tip link consisting of a heterodimer of a protocadherin, PCDH15, and a cadherin, CDH23 (Vollrath et al., 2007). A mechanotransduction complex (Qiu and Müller, 2018; Holt et al., 2021), is present in all but the tallest stereovilli and this complex consists of pore-forming cation channels, TMC1 and/or TMC2, and two other membrane proteins, TMIE and TMHS/LHFPL5 which help modulate the pore properties of the channel [TMIE; (Zhao et al., 2014; Cunningham et al., 2020)] and bind to PCDH15 [TMHS; (Xiong et al., 2012; Zhao et al., 2014; Ge et al., 2018)]. Loss of any of these proteins compromises hair cell function and causes severe hearing loss. Deflection of the hair bundle applies force to each tip link, leading to an extremely fast (∼10 µs) gating of the mechanotransduction channel (Gillespie and Müller, 2009). An array of accessory proteins (such as MYOSIN7A, harmonin, and sans) inside the stereovilli anchors the mechanotransduction complex to the actin core of each stereovillus (Schwander et al., 2010), and mutations in these proteins, or in CDH23 or PCDH15, lead to hereditary deaf-blindness known as Usher syndrome (Cosgrove and Zallocchi, 2014; Whatley et al., 2020). In addition, a second mechanosensitive ion channel, PIEZO2, lies at the base of the hair bundle and is responsible for what have been termed reverse-polarity currents (Beurg and Fettiplace, 2017; Wu et al., 2017), although precise function of PIEZO2 in hair cell mechanotransduction and bundle integrity is still unclear (Qiu and Müller, 2018).

As discussed in Section 3.4, the coronal sensory cells of tunicates show a far greater degree of diversity in different taxa than those of vertebrates (Manni et al., 2006; Caicci et al., 2010a; Rigon et al., 2013). This diversity is seen in the number of cilia, which can vary from just one or two in some groups, to multiple cilia that can be present in single or multiple rows (Table 1; Figure 4B; Figure 6C). The cilia can be located centrally or eccentrically as in vertebrates. Short microvilli can be present or can be elongated to appear more like stereovilli. In most tunicate taxa the stereovilli are of the same length, but in some groups the stereovilli have a more graded morphology reminiscent of a vertebrate hair bundle. Multiple different morphologies of sensory cells can occur in the coronal organs of some taxa, again reminiscent of the different hair cell types seen in vertebrate sensory organs, such as inner and outer hair cells of the mammalian cochlea, type I or type II vestibular hair cells, or the tall and short hair cells of the bird hearing organ, the basilar papilla. Unlike vertebrates, tunicate sensory cells do not appear to have clear tip links connecting their stereovilli, but some taxa show evidence of lateral connections between stereovilli, or between stereovilli and cilia (Burighel et al., 2003; Caicci et al., 2007; Rigon et al., 2013); (Table 1). Such links have some resemblance to the side links, ankle links, shaft connectors and top connectors that are present between stereovilli and between stereovilli and the kinocilium (Richardson and Petit, 2019).

What types of stimulus gate tunicate sensory cells? As discussed in Section 3.5 above, gentle stimulation of the oral tentacles by direct touch, vibration, or electrical shocks can lead to contractions of the atrial and oral siphons known as the crossed response, with stronger stimuli evoking a squirt response caused by strong contractions of both siphons and the body wall (Mackie et al., 2006; Manni et al., 2018). Similar responses can be evoked by particulate matter such as polystyrene beads or ground vegetable matter (Mackie et al., 2006), suggesting that at least one function of the coronal organ is to mediate particle expulsion in response to direct mechanical stimulation. This does not preclude other functions for sensory cells; in this regard it is intriguing that the bundle morphology of some tunicate sensory cells resembles that of electroreceptors seen in many fish and some amphibians (Baker, 2019). Elucidating the types of stimuli that tunicate coronal sensory cells respond to requires more electrophysiology studies such as whole-cell voltage clamp recordings from sensory cells or using fluid jet stimulation to evoke and measure mechanotransduction currents. Additionally, CRISPR may be used to create transgenic tunicates expressing a membrane-localized calcium sensor to detect mechanotransduction and presynaptic activity in hair cells.

It is currently unknown how coronal sensory cells respond to mechanical force, nor the range of forces that can evoke synaptic release. The wide variety of tunicate sensory bundle types, together with the absence of apical tip links in coronal sensory cells suggests it is unlikely that CDH23/PCDH15-mediated gating of a mechanotransduction channel of the sort seen in vertebrates is occurring in tunicates. However, the presence of side links between stereovilli and between stereovilli and cilia suggest an alternative method of mechanoreceptor gating. Indeed, such kinociliary links have been shown to mediate mechanotransduction in developing zebrafish hair cells before being replaced by stereovilli-based mechanotransduction in mature hair cells (Kindt et al., 2012). Insect chordotonal organs facilitate mechanotransduction with members of the TrpN and TrpV channel family (Li et al., 2018). Although vertebrate hair cells seem to use TMC and PIEZO2 channels for mechanotransduction (see above), TrpN channels may also be required for mechanosensation in some cases (Sidi et al., 2003). The TRPA1 channel was originally proposed as candidates for the vertebrate mechanotransduction channel (Corey et al., 2004), but data from knockout mice suggests that neither TRPA1 nor 32 other Trp channels are necessary for mechanotransduction in mouse hair cells (Kwan et al., 2006; Wu et al., 2016). A Ciona orthologue of *TrpA1* is expressed in coronal sensory cells (Rigon et al., 2018), but its role in mechanotransduction has yet to be tested. PIEZO2 is another possible candidate for the tunicate mechanotransduction channel; it is located at the base of the hair bundle in vertebrate hair cells (Wu et al., 2017) and therefore does not require tip-link based mechanotransduction. PIEZO2 also mediates Merkel cell mechanotransduction without the need for elaborate stereovilli or tip link-based machinery (Maksimovic et al., 2014; Woo et al., 2014; Nakatani et al., 2015).

### 4.6 Are vertebrate hair cells and tunicate coronal sensory cells homologous?

During chordate evolution, some cell types remain tightly conserved while others have been either lost or convergently evolved across different species. The concept of a “core regulatory complex” (CoRC) of transcription factors has been useful in devising evolutionary scenarios for cell types (Arendt et al., 2016) and as discussed above, mechanosensory cells across vertebrate and invertebrate taxa appear to share factors such as atonal/Atoh1, senseless/Gfi and Pou4 factors. Several models for the evolution of chordate mechanosensory cells have been proposed (for example, Schlosser, 2021). These models propose some form of basal primary sensory cell giving rise to two distinct cell types: a primary sensory neuron that is not mechanosensitive and defined by neurogenin-like transcription factors, and a mechanosensitive cell defined by atonal-like transcription factors and which either lacked an axon altogether (hair cells and coronal sensory cells) or just a short axon (caudal epidermal neurons; see 2.1 above).

At present, only atonal/Atoh1 expression has been characterized in the tunicate coronal organ and has not yet been localized to the coronal sensory cells. Nevertheless, the presence of both hair cell-like cells adjacent to supporting cells, the expression of Notch pathway genes in these cell types and their derivation from proto-placodal structures make a reasonable case for homology between these cell types. However, this conclusion is complicated by the fact that tunicates undergo metamorphosis, which prevents a clear visualization of the transition from tunicate “proto-placodal” structures to a sensory organ. This transition can be readily observed in vertebrates as the pre-placodal domain gives rise to individual placodes, some of which produce hair cells.

Resolving the question of homology between vertebrate hair cells and tunicate coronal sensory cells will be helped by three recent technical advances. First, single cell transcriptional analysis will be able to determine whether the CoRC transcription factors present in vertebrate hair cells and supporting cells are also expressed in coronal sensory cells and their associated supporting/accessory cells. Second, the advent of CRISPR has facilitated loss-of-function studies in many new model and non-model organisms, and disruption of tunicate CoRC mechanosensory transcription factors will allow testing of their necessity for coronal sensory cell differentiation. Finally, it may be possible to perform lineage tracing experiments to determine tunicate proto-placodal cells do indeed contribute to coronal sensory cells following metamorphosis. Resolving these questions could elucidate the ancestral mechanosensory hair cell gene regulatory network or could uncover novel mechanisms of creating mechanosensitive hair cell-like cells in different species.
